# Cancer Differentiation Inducer Chlorogenic Acid Suppresses PD-L1 Expression and Boosts Antitumor Immunity of PD-1 Antibody

**DOI:** 10.7150/ijbs.83599

**Published:** 2024-01-01

**Authors:** Rui Li, Yun Zhan, Xiao Ding, Jinjin Cui, Yanxing Han, Jinlan Zhang, Jie Zhang, Wenbin Li, Lulu Wang, Jiandong Jiang

**Affiliations:** 1 Institute of Medicinal Biotechnology, Chinese Academy of Medical Sciences and Peking Union Medical College, Beijing, 100050, China.; 2 State Key Laboratory of Bioactive Substances and Function of Natural Medicine, Institute of Materia Medica, Chinese Academy of Medical Sciences and Peking Union Medical College, Beijing, 100050, China.; 3 State Key Latoratory of Phytochemistry and Plant Resource in West China, Kunming Institute of Botany, Chinese Academy of Sciences, Kunming, Yunnan, 650201, China.; 4 Jiuzhang Biochemical Engineering Science and Technology Development Co., Ltd, Chengdu, Sichuan, 610041, China.; 5 Department of Neuro-oncology, Cancer Center, Beijing Tiantan Hospital, Capital Medical University, Beijing 100070, China.

**Keywords:** Chlorogenic acid, Cancer differentiation inducer, PD-L1, Anti-PD-1 antibody, Combination therapy

## Abstract

As immune checkpoint inhibitors have shown good clinical efficacy, immune checkpoint blockade has become a vital strategy in cancer therapy. However, approximately only 12.5% patients experience benefits from immunotherapy. Herein, we identified the cancer differentiation inducer chlorogenic acid (CGA, now in the phase II clinical trial in China for glioma treatment) to be a small-molecular immune checkpoint inhibitor that boosted the antitumor effects of the anti-PD-1 antibody. CGA suppressed the expression of PD-L1 induced by interferon-γ in tumor cell culture through inhibition of the p-STAT1-IRF1 pathway and enhanced activity of activated T-cells. In two murine tumor xenografts, combination therapy of CGA with anti-PD-1 antibody decreased the expression of PD-L1 and IRF1 and increased the inhibitory effect of the anti-PD-1 antibody on tumor growth. Particularly, the activity of tumor infiltrated T cells was enhanced by CGA. CGA improved the gene expression of granzymes in tumor-infiltrated immune cells. In conclusion, through induction of differentiation, CGA appeared to suppress the expression of PD-L1 on cancer cells, effectively promoting infiltrated T cells in the tumor and boosting the antitumor effect of the anti-PD-1 antibody. Thus, CGA might serve as a promising agent to enhance anticancer immunotherapy if combined with anti-PD-1 antibodies.

## Introduction

Immune checkpoint is an important mechanism to avoid autoimmune response and keep immune system in homeostasis. However, activation of immune checkpoint is also one of the main mechanisms for tumor survival in cancer patients. Initiation of checkpoint pathways results in the exhaustion of cytotoxic T lymphocytes, which subsequently takes the tumor cells to bypass immune surveillance. Programmed cell death 1/ programmed cell death ligand 1 (PD-1/PD-L1) axis is one of the well-known pathways serving the immune checkpoint function. PD-L1 has been reported to highly expressed in cancers such as melanoma, lung, breast, ovarian, pancreas and colon cancer 1-3. As an immune checkpoint molecule, the function of PD-L1 is to interact with its receptor PD-1 that expressed on the surface of tumor-infiltrating T-lymphocytes, causing inhibition of cytotoxic T-cells activation and generating collapse of immune surveillance to cancer. In the past decades, antibodies against immune checkpoint molecules have become a hot research focus, in attempt to interrupt the immune checkpoint function and to discover drugs for cancer immunotherapy.

The first immune checkpoint inhibitor (ICI) is ipilimumab, which is an antibody for cytotoxic T lymphocyte-associated protein 4 (CTLA-4) and clinically effective in treating metastatic melanoma. Then, antibodies for PD-1 (pembrolizumab and nivolumab) and its ligand PD-L1 (atezolizumab, durvalumab, and Avelumab) have been approved, and widely used for curbing different kinds of cancers 4. Moreover, clinical trials for novel ICIs with similar mode of action or for new applications to various cancers are being tested in hospitals worldwide. However, although with great successes, the expected anticancer immune response was only seen in some 12.5% of patients 5, 6. The difference of anticancer immune response observed in patients has been attributed to a variety of reasons, such as expression levels of PD-L1 and PD-1, genetic mutations of cancer cells, as well as development of neo-antigens 7, and represents a grand challenge to cancer immunotherapy. Investigation on biomarkers to identify suitable patients for the ICI antibody treatments has been considered one of the approaches to avoid ineffective use of ICIs in patients 8, 9. Combination of ICI antibodies with conventional anticancer agents is another attempt to promote treatment outcome 10. One example is the combination therapy using anti-PD-1 antibody together with gemcitabine, which enhanced the anticancer effect of the antibody through activation of macrophages and CD8+ T cells 11, 12, and, the enhancing effect by gemcitabine was independent of PD-L1 expression from the tumor cells 13. Although either monotherapy with PD-1 inhibitor or their combination therapies have achieved notable success in clinic, the low response rate to PD-1 inhibitor remains to be a big concern 14. It is obvious that mechanism-based rational designs for an optimized ICIs treatment is highly desirable.

Induction of cancer differentiation is our new strategy to transfer cancer cell from a highly invasive and metastatic phenotype to a less malignancy or nearly normal state. By principle in biology, PD-L1 expression in cancer cells should be positively associated with tumor malignancy, as it successfully creates a machinery for immune escape. Recent research has shown that PD-L1 expression appeared to link with poor differentiation in cancer cells or/and stemness 15-20. Sun and colleagues indicated that mesenchymal stem cells (MSCs) induce PD-L1 expression in gastric cancer cells via STAT3/mTOR-c-Myc signal axis 19. Wang *et al* demonstrated that PD-L1-mediated immune escape was related to the activation of c-Myc and EGFR/MAPK signaling pathways in non-small cell lung cancer 20. Liang and colleagues demonstrated that c-Myc could induce the expression of PD-L1 in esophageal squamous cell carcinoma 21, while others showed that PD-L1 might enhance c-Myc activity in lung adenocarcinoma 15. These findings suggested an interesting correlation between tumor differentiation and immune checkpoint in cancers. Thus, we hypothesized that an improved differentiation status in tumor cells might reduce their PD-L1 production, thus enhance the activity of cytotoxic T-cells in tumor microenvironment. If true, cancer differentiation inducers might promote anticancer activity of ICIs, additional to their own anticancer effect.

Chlorogenic acid (CGA), a polyphenol compound and an over-the-counter (OTC) drug (oral) for inflammation in China, has been reported to have multiple pharmacological activities such as anti-oxidant, anti-inflammatory and neuroprotection 22. Recent evidence has demonstrated that CGA could suppress the proliferation of various cancer cells 23-26. Our Phase I clinical trial (using the intramuscular injection formula) showed that CGA was very well tolerated and demonstrated a significant overall survival benefit for the patients with recurrent high-grade glioma 27. Based on the results, China FDA has approved CGA for phase II clinical trials in cancer patients in 2017 (Phase II. NCT 2013L01855). For its mode of action, our previous work has acknowledged that CGA inhibited the tumor growth of hepatoma and lung cancer via inducing differentiation in cancer cells. The expression of genes associated with poor differentiation, such as c-Myc, was mainly downregulated by CGA, through upregulation of SUMO1 expression and c-Myc sumoylation, leading to a strong suppression of c-Myc and maturation phenotype in cancer cells 28. We have also found that CGA down-regulated the expression of BMI1 and SOX2, the tumor-associated stem cell markers, in a dose- and time- dependent manner in esophageal squamous cell carcinoma 29. In the present study, we utilize CGA to show that induction of tumor differentiation could down-regulate cancer PD-L1 expression, activate cytotoxic T lymphocytes in tumor tissues, and boost immunotherapy of currently available ICI antibodies in combination. As CGA is safe in human, the presented discovery might be quickly translated into clinical use.

## Results

### CGA decreased PD-L1 expression induced by IFN-γ in cancer cell lines

**Figure 1:**
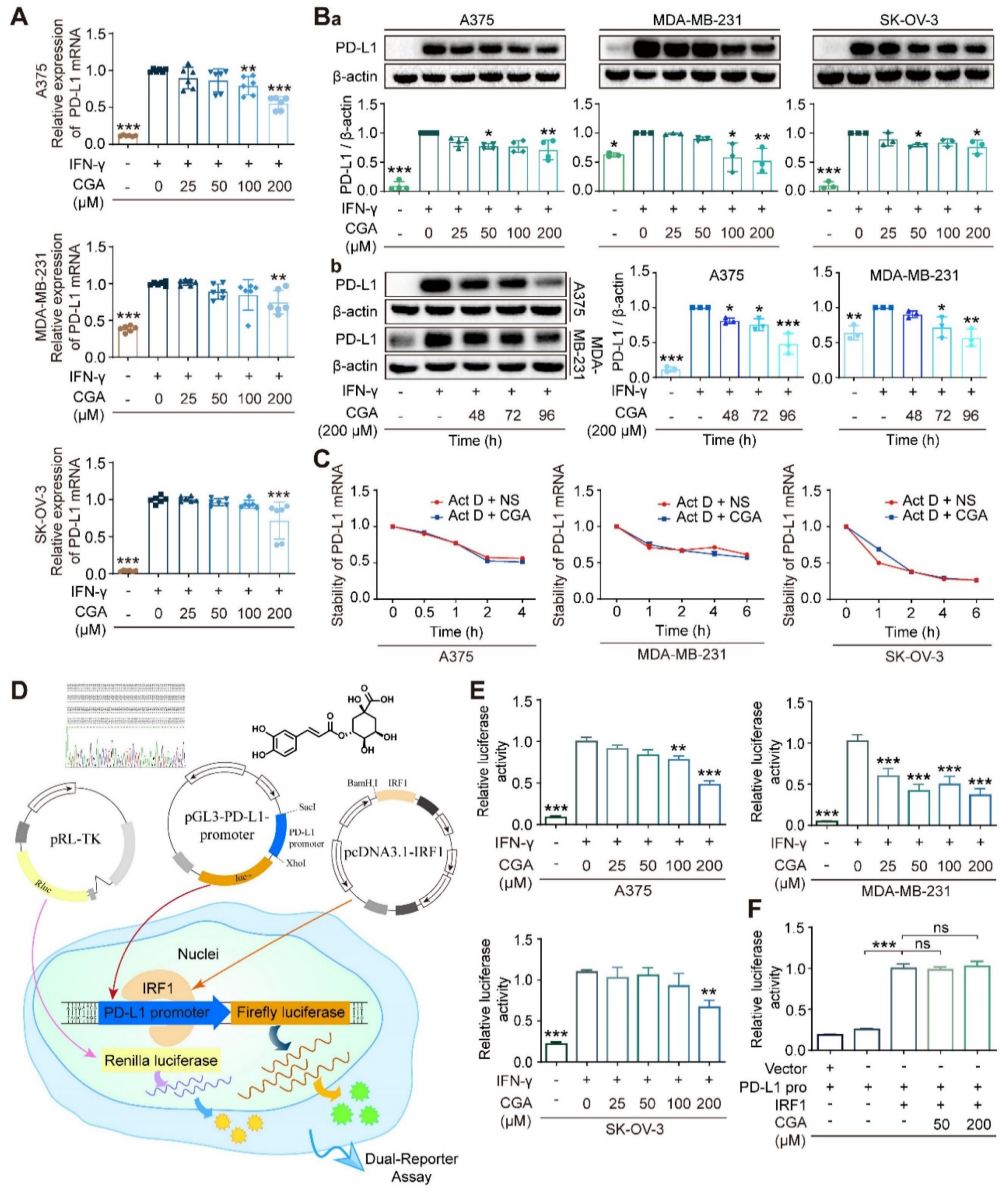
**CGA suppressed PD-L1 expression of cancer cells.** CGA diminished PD-L1 expression induced by IFN-γ in a variety of cancer cells. Human melanoma cell line A375, ovarian cancer cell line SK-OV-3, and breast cancer cell line MDA-MB-231 were treated with IFN-γ (10 ng/mL) and CGA (0, 25, 50, 100, 200 μM) for 48 h at 37˚C in 5% CO2. The mRNA expression of PD-L1 was evaluated by RT-PCR. The results were normalized to GAPDH (**A**). The protein expression of PD-L1 was tested using Western blot analysis. The results were normalized to β-actin as density ratio (**B**). The PD-L1 protein expression was influenced through a dose- (Ba) and time-dependent (Bb) fashion after CGA administration. **C**. The stability of PD-L1 mRNA w.as not influenced by CGA treatment. The cells were treated with IFN-γ (10 ng/mL) and CGA (200 μM). Act D (5 μM) were added to disrupt the stability of PD-L1 mRNA. The mRNA of PD-L1 was evaluated by RT-PCR at 0, 1, 2, 4, 6 hrs in SK-OV-3 and MDA-MB-231 cell lines while at 0, 0.5, 1, 2, 4 hrs in A375 cell line. The results were normalized to GAPDH. **D-F.** CGA reduced the activation of the PD-L1 promoter by suppressing IRF1 expression. The A375, SK-OV-3, and MDA-MB-231 cells were transfected with pGL3-PD-L1-promoter plasmid (100 ng per well) and pRL-TK normalization plasmid (3 ng per well) for 6 hrs, and treated with CGA (0 - 200 μM) and IFN-γ (10 ng / mL) for another 48 hrs (**D**). At the end of the experiment, cells were treated with 100 μL of PLB and the lysate was measured using the Dual-Luciferase ® Report Assay System and a GloMax 96 Microplate Luminometer (Promega). The activity of PD-L1 promoter was calculated by Relative luciferase units (RLUs), normalized to Renilla luciferase signal (**E**). The plasmid of IRF1 was co-transfected with pGL3-PD-L1-promoter and pRL-TK plasmids into HEK-293T cells. The activity of PD-L1 promoter was were calculated by Relative luciferase units (RLUs), normalized to Renilla luciferase signal (**F**). Data are presented as mean ± SD (n = 6, 3 or 4). Significant differences are indicated: *p< 0.05, **p< 0.01, ***p< 0.001, ns, not significant, vs. 0 μM group by One-way ANOVA test. CGA indicated chlorogenic acid; IFN-γ, interferon-γ; IRF1, interferon regulation factor 1.

In this study, 7 cancer cells (5 human cancer cell lines and 2 murine cancer cell lines) were agitated by IFN-γ and then treated with CGA; among the 7 cell lines human melanoma (A375), ovarian cancer (SK-OV-3), and triple-negative breast cancer (MDA-MB-231) cells were reported to have basic PD-L1 expression. As shown in **Fig. 1A**, as compared with the untreated, CGA significantly reduced PD-L1 expression induced by IFN-γ (10 ng/ml) in these three cancer cell lines (200 μM; 48 hrs; p < 0.001, 0.01, 0.001, respectively). This effect was then confirmed at the protein level (p < 0.01, p < 0.01, p < 0.05, respectively) (**Fig. 1Ba**). Similar phenomena were also seen in murine colon carcinoma cell MC38 and murine breast cancer cell 4T1 (**Supplementary Fig. S1**). However, no significant difference was found in human lung large cell carcinoma cell NCI-H460 and squamous cell carcinoma of head and neck cell CAL-27 (**Supplementary Fig. S2**). The results indicated that the inhibitory effect of CGA on PD-L1 expression might be different among cancer cell lines. We should mention here that the safety of CGA at 200 μM has been very well confirmed previously 28. The well-known differentiation inducer retinoid acid (RA) for leukemia was tested also as a reference. As shown in **Supplementary Fig. S3**, in the three tested cell lines, RA inhibited IFN-γ-induced PD-L1 expression in the SK-OV-3 cell line, but increased the PD-L1 expression in the A375 and MDA-MB-231 cell lines. Thus, RA's activity on PD-L1 expression remains further studies.

Induction of cancer differentiation is a complicated process and takes a period. Therefore, PD-L1 expression in extended incubation (48, 72, 96 hrs) of cancer cells were tested, in the presence or absence of CGA. As shown in **Fig. 1Bb**, the inhibition of PD-L1 protein expression by CGA in the extended incubation (72 hrs and 96 hrs) were more profound as compared to that that in the 48 hrs culture, in either A375 or MDA-MB-231cell lines (CGA 200 μM; vs untreated; for A375, p < 0.05, p < 0.001; for MDA-MB-231, p < 0.05, p < 0.01), with null of obvious cytotoxicity. It appears to us that the declined PD-L1 expression might be part of the cell response to the differentiation induction by CGA.

### CGA decreased PD-L1 expression through suppressing p-STAT1/STAT1-IRF1-PD-L1 promoter pathway

To elucidate the mechanism, we firstly evaluated the stability of PD-L1 mRNA, from which we found that the stability of PD-L1 mRNA was not influenced by CGA (**Fig. 1C**). Then, we detected the effect of CGA on the activity of the PD-L1 promoter. Plasmids containing PD-L1 promoter with Firefly luciferase reporter were constructed and co-transfected with pRL-TK into three cancer cell lines. The experimental schematic is in **Fig. 1D**. As shown in **Fig. 1E**, IFN-γ increased the activity of the PD-L1 promoter (p < 0.001), but the stimulus was suppressed by CGA intervention (200 μM) in all three cancer cell lines (p < 0.001, p < 0.001, p < 0.01). Then, we co-transfected the PD-L1 promoter and the IRF1 expression plasmid into the HEK-293T cells, which revealed that the inhibitory effect of CGA on the PD-L1 promoter was diminished by the overexpression of IRF1 (**Fig. 1F**), suggesting that suppression of PD-L1 expression by CGA might be mediated through inhibiting the transcription factor IRF1.

**Figure 2:**
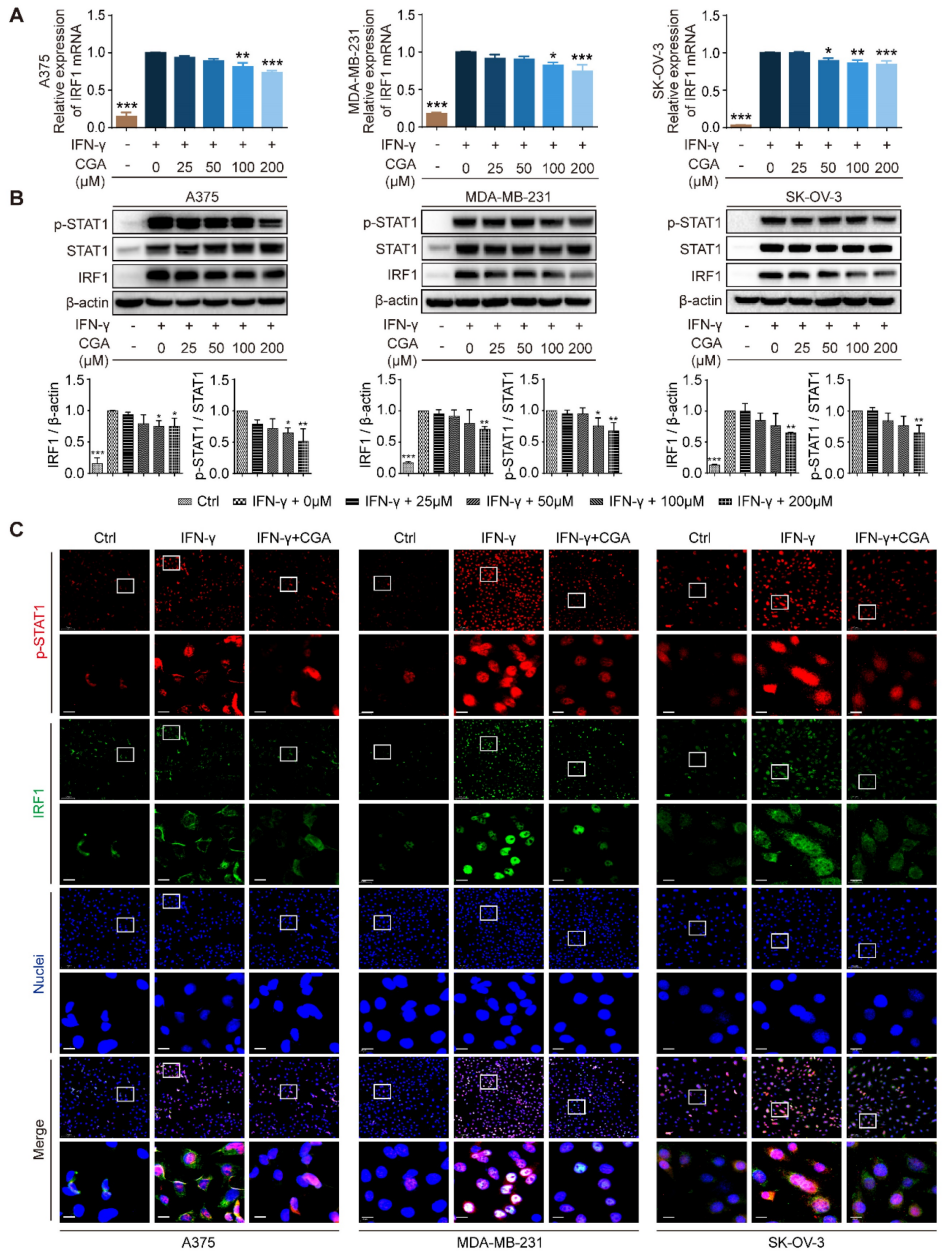
** Inhibition of STAT1 phosphorylation by CGA lead to a suppression of IRF1 expression.** Human melanoma cell line A375, ovarian cancer cell line SK-OV-3, and breast cancer cell line MDA-MB-231 were treated with IFN-γ (10 ng/mL) and CGA (0, 25, 50, 100, 200 μM) for 48 hrs at 37˚C in 5% CO2. **A**. The mRNA expression of IRF1 was evaluated by RT-PCR. The results were normalized to GAPDH.** B**. The protein expression of IRF1 and phosphorylation of STAT1 (p-STAT1/STAT1) were tested using Western blot analysis. The results of IRF1 were normalized to β-actin, and the phosphorylation levels of STAT1 were normalized to STAT1 as density ratio. **C**. The representative images of multi-color immunofluorescent staining for p-STAT1 (red) and IRF1 (green) in A375, MDA-MB-231, and SK-OV-3 cell lines. The regions of interest (ROI) are boxed in white, and their magnified photos are shown below. Scale bars, 10 µm. Data are presented as mean ± SD (n = 3). Significant differences are indicated: *p< 0.05, **p< 0.01, ***p< 0.001, vs. 0 μM group by One-way ANOVA test. Ctrl indicated control; STAT1, signal transducer and activator of transcription 1; p-STAT1, phosphorylated signal transducer and activator of transcription 1.

The JAK-STAT1-IRF1 pathway links with IFN-γ induced upregulation of PD-L1, upstream of which IFN-γ binds the IFN-γ receptor (IFNGR1 and IFNGR2) and then activates JAK-STAT1^30^. In the present study, the expression of IFNGR1 and IFNGR2 was examined, and showed no changes after CGA treatment (**Supplementary Fig. S4**). We found that CGA downregulated the expression of IRF1 at mRNA and protein levels (**Fig. 2A-B**), but the stability of IRF1 mRNA was not changed by CGA treatment (**Supplementary Fig. S5**). Next, the levels of STAT1 and phosphorylated STAT1 were evaluated, which revealed that IFN-γ induced STAT1 expression and phosphorylation augments were significantly reversed by CGA in these three cancer cell lines. Immunofluorescence assay confirmed that the expression of p-STAT1 and IRF1 were largely attenuated by CGA (**Fig. 2C**). Furthermore, when the STAT1 expression induced by IFN-γ was knocked down with siRNAs, the inhibition of PD-L1 expression by CGA was significantly diminished (**Supplementary Fig. S6**). Additionally, the basal level of PD-L1 was not changed by CGA treatment in the absence of IFN-γ (**Supplementary Fig. S7**). In short, the results indicated that CGA suppressed phosphorylation of STAT1, resulting in an inhibition of IRF1 and subsequent attenuation of the activity of PD-L1 promoter, leading to diminished PD-L1 expression.

### CGA enhanced the sensitivity of cancer cells to the co-cultured T cells

**Figure 3:**
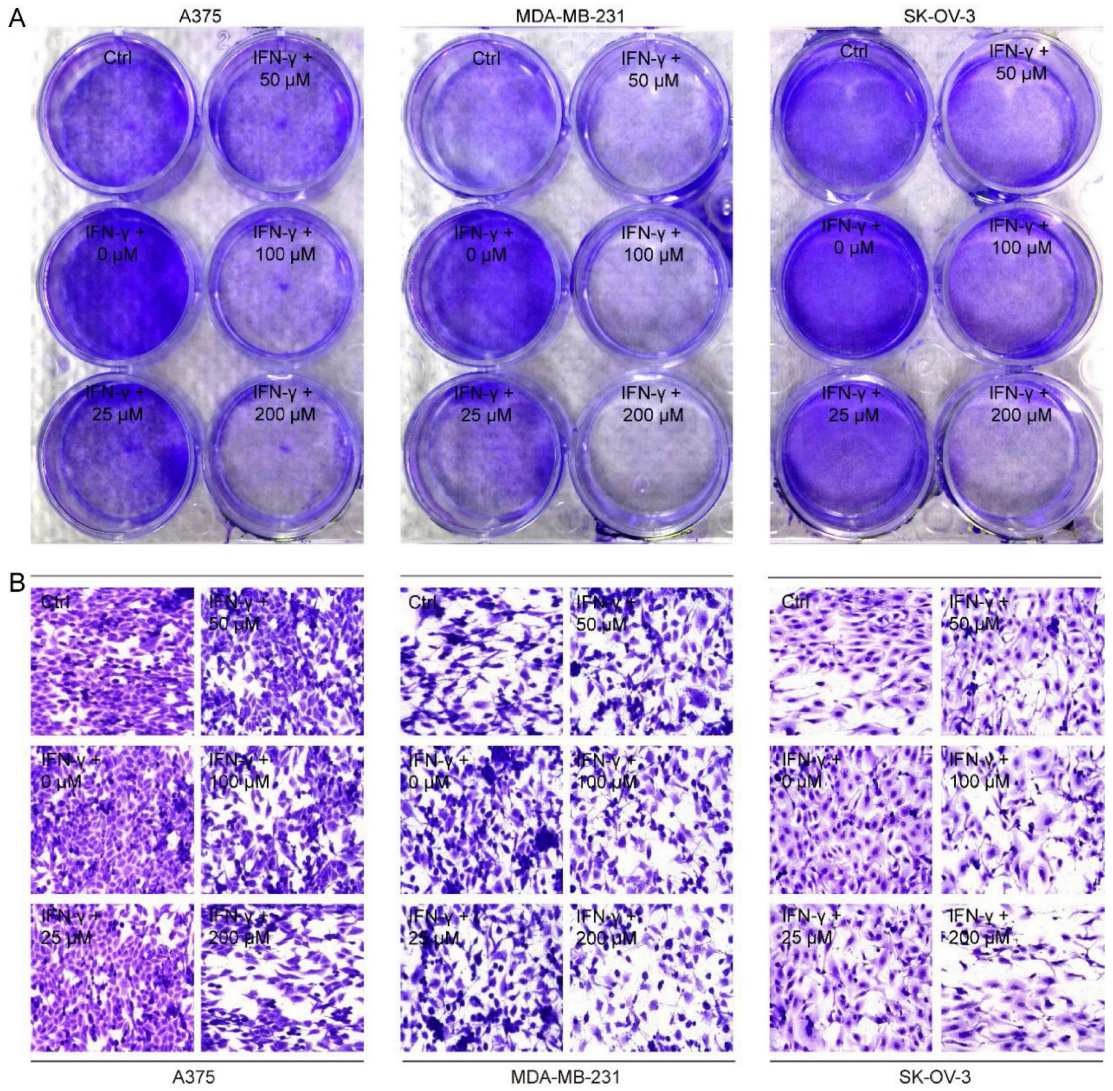
**CGA enhanced the sensitivity of cancer cells to the co-cultured T cells.** Human melanoma cell line A375, ovarian cancer cell line SK-OV-3, and breast cancer cell line MDA-MB-231 were treated with IFN-γ (10 ng/mL) and CGA (0, 25, 50, 100, 200 μM) for 24 hrs at 37˚C in 5% CO2. Human T lymphocytic leukemia cells Jurkat E6 were treated with anti-CD3 antibody (100 ng/mL) and anti-CD28 antibody (100 ng/mL) for 24 hrs at 37˚C in 5% CO2. The cancer cells and activated Jurkat E6 cells were co-cultured at the ratio of 1:5 for 24 hrs. The survived tumor cells were stained with crystal violet (**A**) and observed by inverted microscope (**B**). Representative images of crystal violet staining (400×).

Next, we co-cultured A375, MDA-MB-231 and SK-OV-3 cells, respectively, with PD-1 expressed activated Jurkat E6 T cell to imitate the tumor cell killing effect by T cells when PD-1 and PD-L1 interacted (**Supplementary Fig. S8**), and then examined the cancer cell proliferation after exposure to CGA and IFN-γ. As shown in **Fig. 3A**, Jurkat E6 T cell significantly reduced the proliferation of co-cultured cancer cells without any treatment, while IFN-γ (10 ng/mL) significantly attenuated T cells killing efficacy and facilitated proliferation of cancer cells through increased PD-L1 expression in the co-cultured system. Interestingly, CGA (200 μM) treatment restored the anticancer activity and increased killing efficacy of the activated T cells (**Fig. 3B** and** Supplementary Fig. S9**). These results suggested that CGA strengthened the anticancer ability of activated T cells for the co-cultured cancer cells, via its inhibition on PD-L1 expression in the cell lines.

### Combination of CGA with anti-PD-1 antibody significantly increased suppression on tumor growth in two xenografts mice model

**Figure 4:**
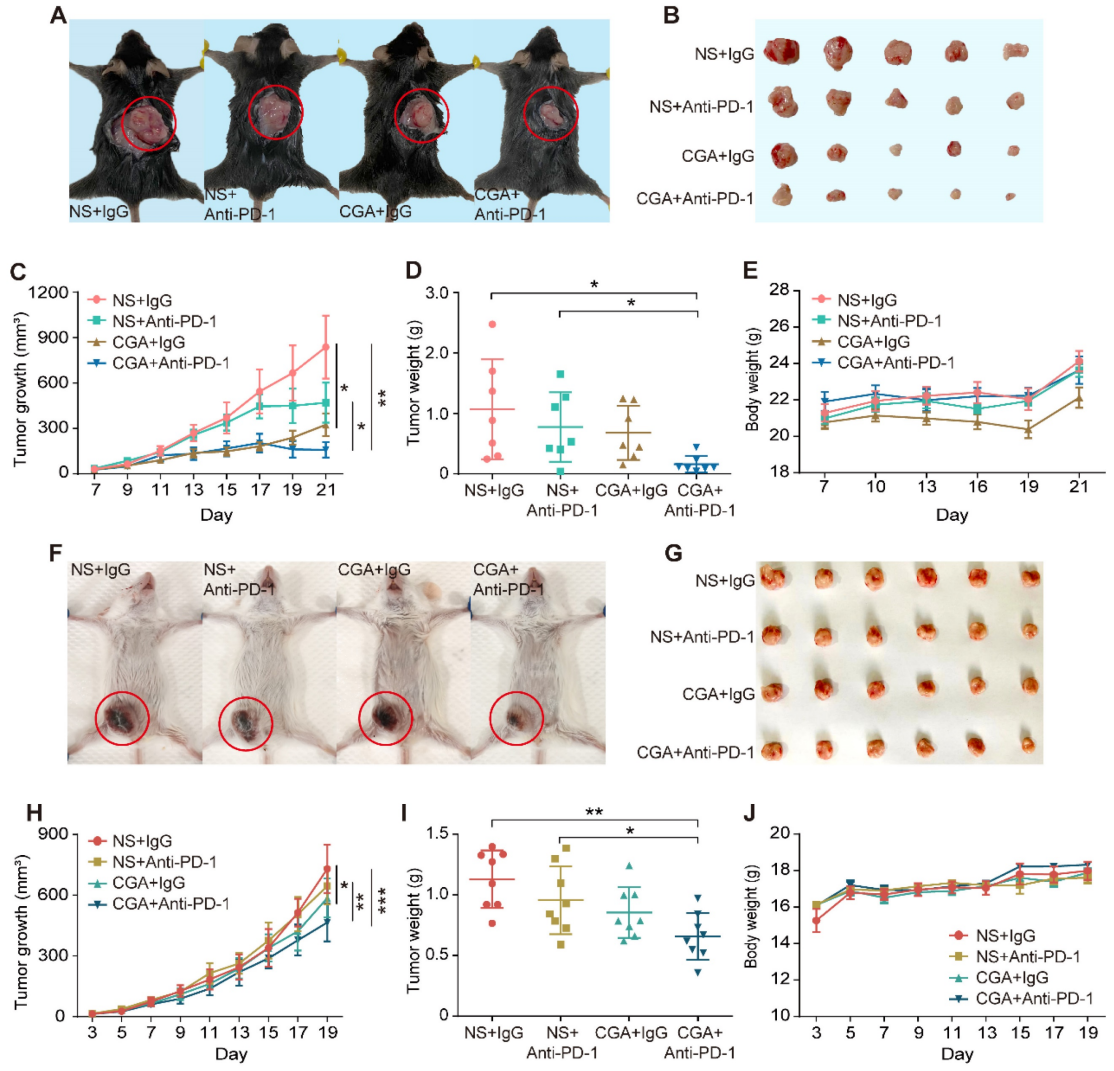
**CGA strengthened the anti-tumor effect of anti-PD-1 antibody *in vivo*. A-E**. CGA improved the anti-tumor effect of anti-PD-1 antibody in murine colon cancer. 2 × 10^5^ of murine colon carcinoma MC38 cells were injected under the skin of the back of male C57BL/6N mice. Three days after inoculation, the mice were randomly divided into four groups (normal saline combined with IgG: NS + IgG, normal saline combined with anti-PD-1 antibody: NS + Anti-PD-1, CGA combined with IgG: CGA + IgG, and CGA combined with anti-PD-1 antibody: CGA + Anti-PD-1) (n = 7). The CGA (50 mg / kg) was administrated (i.p.) once a day for three successive weeks, while the antibodies (200 ug) were given at the 3rd, 7th, and 10th day, respectively. **A**. The representative image of tumor-load mice. The tumors are circled in red. **B**. The representative images of excised tumors. **C**. Diagram of tumor growth. **D**. Comparison of the weight of the tumors from the mice in each group. **E**. The change of body weight. **F-J**. CGA improved the anti-tumor effect of anti-PD-1 antibody in murine breast cancer. 1 × 10^5^ of murine breast cancer cell 4T1 were injected into the mammary fatty pad of the female BALB/c mice. Three days after inoculation, the mice were randomly divided into four groups described above. The CGA (50 mg / kg) was administered (i.p.) once a day for 19 successive days, while the antibodies (200 ug) were given at the 3rd, 7th, and 10th day, respectively. **F**. The representative image of tumor-load mice. The tumors are circled in red. **G**. The representative images of excised tumors. **H**. Diagram of tumor growth. **I**. Comparison of the weight of the tumors from the mice in each group. **J**. The changes of body weight. Data shown are mean value ± SD. Significant differences are indicated: *p< 0.05, **p< 0.01, ***p< 0.001, vs. NS + IgG group by One-way ANOVA test. NS indicated normal saline; IgG, isotype.

Antitumor effects of anti-PD-1 antibody, CGA, and their combination (Anti-PD-1 antibody, 200 μg per injection, i.p. at 3^rd^, 7^th^, 10^th^ day post tumor innoculation; CGA, 50 mg/kg, i.p. for 21 days and 19 days) were examined by measuring tumor growth suppression in two xenografts mice models (**Fig. 4A-B and 4F-G**). As shown in **Fig. 4C**, combination treatment dramatically suppressed the tumor growth in the MC38 tumor xenografts with an inhibition rate of 81.3% (CGA + Anti-PD-1 vs. NS + IgG, n = 7, p < 0.01). Similar results were seen in the 4T1 tumor xenografts (**Fig. 4H**), although the inhibition rate was only 36.2% (CGA + Anti-PD-1 vs. NS + IgG, n = 8, p < 0.001). Accordingly, the tumor weights in the two models significantly declined as well after combination therapy (CGA + Anti-PD-1 vs. NS + IgG, p < 0.05 for MC38, p < 0.01 for 4T1) (**Fig. 4D and 4I**). It was obvious that the combination therapy showed a superior antitumor effect over the anti-PD-1 antibody did in both tumor growth and tumor weights (CGA + Anti-PD-1 vs. NS + Anti-PD-1, p < 0.05, p < 0.05 for MC38; p < 0.01, p < 0.05 for 4T1). During the treatment period, monotherapy with CGA or anti-PD-1 antibody, or their combination did not influence the body weight of the mice, suggesting a good safety of the agents (**Fig. 4E and 4J**). It appeared that addition of CGA significantly boosted the anticancer efficacy of the anti-PD-1 antibody. Explanations for the different tumor suppression efficacy of the combination therapy in the MC38 and 4T1 tumor in mice were discussed in the discussion part.

### The enhanced anti-tumor effect of combination therapy might be mediated through a CGA-caused down-regulation of PD-L1 expression in tumor

**Figure 5:**
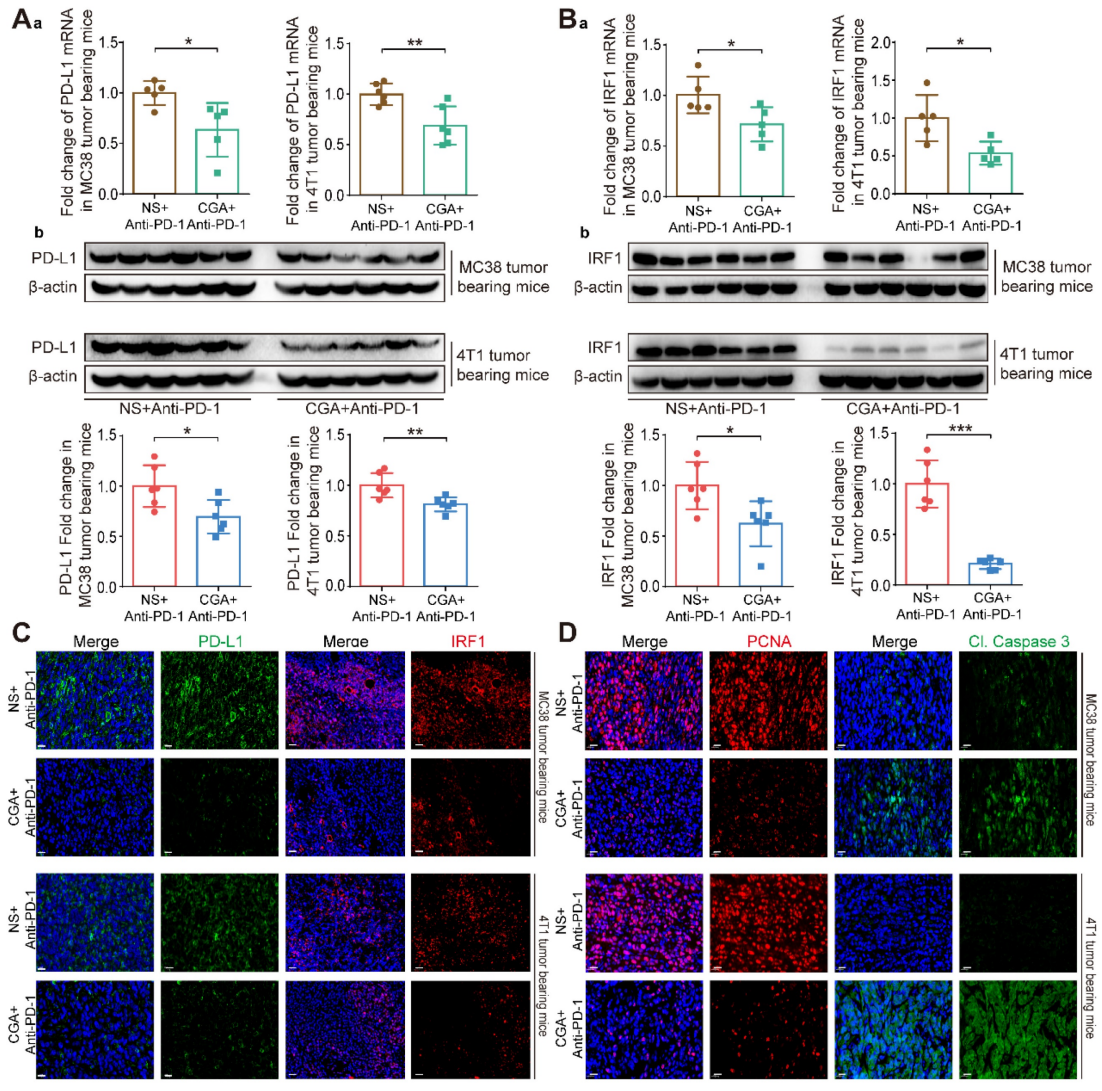
** CGA enhanced the anti-tumor effect of anti-PD-1 antibody monotherapy through the inhibition of PD-L1**. The tumor tissues were collected from mice in anti-PD-1 antibody monotherapy group (NS+Anti-PD-1) and CGA+Anti-PD-1 antibody combination treatment group (CGA+Anti-PD-1). At the end of the experiment, the tumors tissues were collected, and the total RNA and protein were extracted. **Aa**. The mRNA expression of PD-L1 in tumor tissues was evaluated by RT-PCR. The results were normalized to GAPDH. **Ab**. The protein expression of PD-L1 was tested by Western blot analysis. The results of PD-L1 were normalized to β-actin, as density ratio. **Ba**. The mRNA expression of IRF1 in tumor tissues was evaluated by RT-PCR. The results were normalized to GAPDH. **Bb**. The protein expression of IRF1 was tested by Western blot analysis. The results of IRF1 were normalized to β-actin, as density ratio. **C**. Representative multi color immunofluorescence images stained of PD-L1 (green) and IRF1 (red) in tumor tissues. Scale bar, 20 μm. **D**. Representative multi color immunofluorescence images stained of PCNA (red) and Cl. Caspase 3 (green) in tumor tissues. Scale bar, 20 μm. Data are presented as mean ± SD (n = 5 or 6). Significant differences are indicated: *p< 0.05, **p< 0.01, ***p< 0.001, vs. NS + Anti-PD-1 group by Student's *t*-test.

We then compared the PD-L1 expression in tumor tissues between the anti-PD-1 antibody group and combination one. As shown in **Fig. 5A**, CGA dramatically down-regulated tumor PD-L1 expression at both mRNA (**Fig. 5Aa**) and protein (**Fig. 5Ab**) levels. PD-L1 mRNA expression was inhibited by 36.5% (CGA +Anti-PD-1 vs. NS + Anti-PD-1, p < 0.05) in the MC38 tumor tissues and 31.0% (CGA +Anti-PD-1 vs. NS + Anti-PD-1, p < 0.01) in the 4T1 tumor tissues. And the PD-L1 protein expression was decreased by 30.5% (CGA +Anti-PD-1 vs. NS + Anti-PD-1, p < 0.05) and 18.9% (CGA +Anti-PD-1 vs. NS + Anti-PD-1, p < 0.01) in the MC38 and 4T1 tumor tissues, respectively. Downregulation of IRF1 (for both mRNA and protein) in tumor tissues by CGA in the combination group was also more effective than that by anti-PD-1 antibody monotherapy (**Fig. 5B,** CGA +Anti-PD-1 vs NS + Anti-PD-1, p < 0.05, p < 0.05 for the MC38 and 4T1 tumor tissues at mRNA level, and p < 0.05, p < 0.001 for MC38 and 4T1 tumor tissues at protein level). The inhibition of PD-L1 and IRF1 in combination therapy was verified with multi-color immunofluorescence assay in tumor tissues (**Fig. 5C and Supplementary Fig. S10**). It suggested that CGA could increase antitumor effect of anti-PD-1 antibody through decreasing the expression of PD-L1 and IRF1. Also, multi-color immunofluorescence assay revealed that the combination therapy inhibited the expression of PCNA, a biomarker of tumor cell proliferation, and promoted Cleaved Caspase 3 (Cl. Caspase 3) expression in two xenografts mice models (**Fig. 5D and Supplementary Fig. S10**). In addition, we evaluated the serum IFN-γ concentration to learn whether the combination therapy suppressed IFN-γ level in blood, and found that the combination therapy did not change IFN-γ concentration in the serum (**Supplementary Fig. S11**). These results suggested that the superior effect of the combination therapy over that of the anti-PD-1 antibody monotherapy could be attributed to the downregulation of PD-L1, at least in part.

### Increase of the activated tumor-infiltrating T-cells and granzymes' genes expression by CGA in tumor tissue

**Figure 6:**
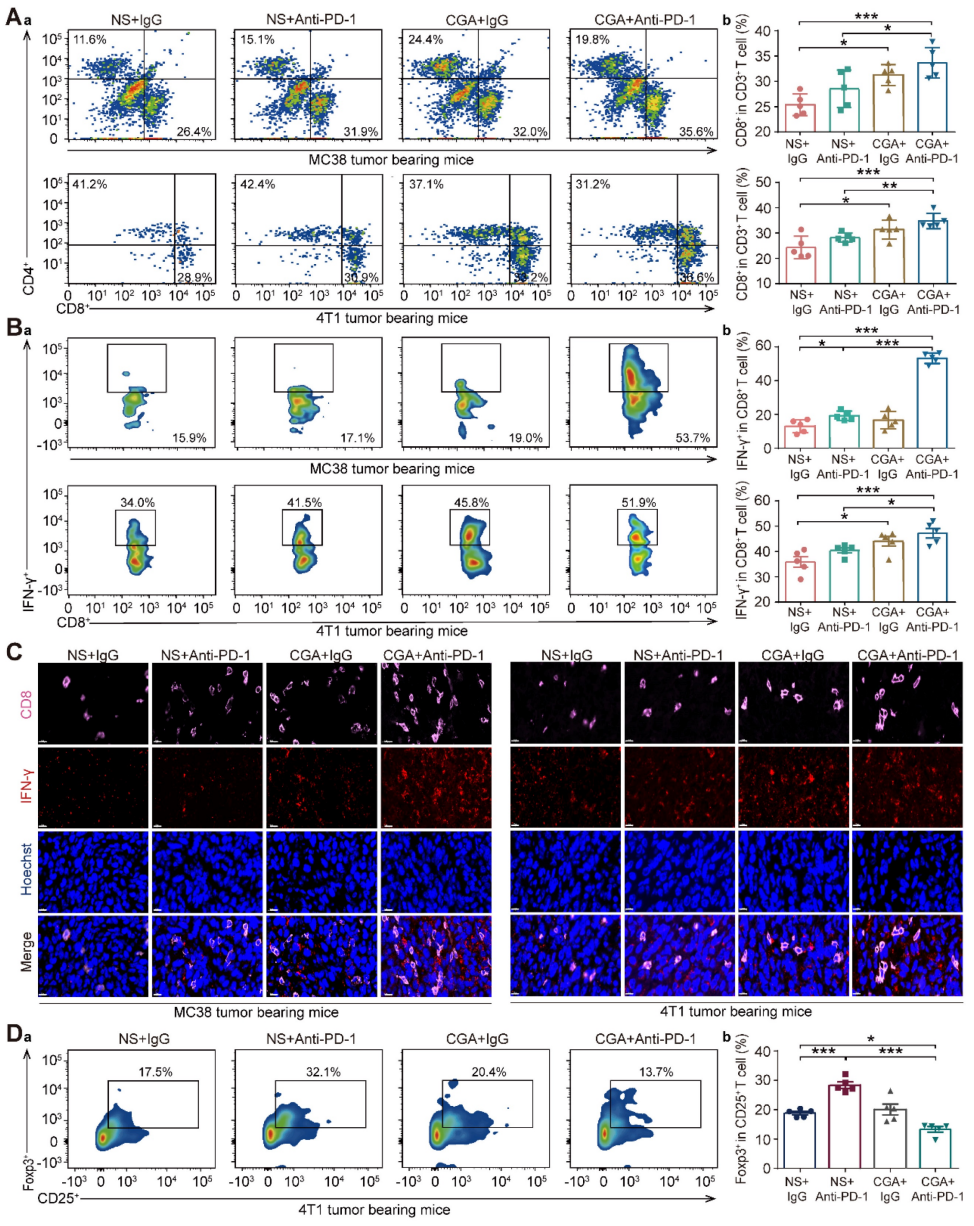
** CGA boosted the infiltration of cytotoxic T lymphocyte in tumor tissues**. Cytotoxic T lymphocyte (CTLs) were isolated from fresh tumor tissues and dissociated into single cell suspension to go through flow cytometry analysis. **Aa**. Representative flow cytometer profiles of infiltrated cytotoxic T cells (CD8^+^ CD3^+^). **Ab**. Percentage of infiltrated cytotoxic T cells. **Ba**. Representative flow cytometer profiles of infiltrated effector T cells (IFN-γ^+^ CD8^+^ CD3^+^). **Bb**. Percentage of infiltrated effector T cells. **C**. Representative multi-color immunofluorescent images stained of CD8 (pink) and IFN-γ (red) of tumor tissues. Scale bar. 10 μm. **Da**. Representative flow cytometer profiles of infiltrated Treg cells (Foxp3^+^ CD25^+^ CD4^+^) in the tumor tissue of 4T1 tumor bearing mice. **Db**. Percentage of infiltrated Treg cells. Data are presented as mean ± SD (n = 5). Significant differences are indicated: *p< 0.05, **p< 0.01, ***p< 0.001, vs NS + IgG. group by One-way ANOVA test.

Cytotoxic T lymphocyte (CTLs) play an essential role in immune response against tumor. As shown in **Fig. 6A-B**, in both models the population of CD8^+^ CD3^+^ T cells and IFN-γ^+^ CD8^+^ CD3^+^ T cells (activated CD8^+^ T cells) in tumor tissues of the mice treated with the drug combination were significantly higher than that in the control (NS + IgG) or anti-PD-1 antibody (NS + Anti-PD-1) groups (for MC38, p <0.001, p < 0.001 vs NS + IgG; and p < 0.05, p < 0.001 vs NS + Anti-PD-1; for 4T1, p < 0.001, p < 0.001 vs NS + IgG, and p < 0.01, p < 0.05 vs NS + Anti-PD-1), indicating that combination of CGA with anti-PD-1 antibody enhanced the activated cytotoxic T-cell in tumor tissue in the tumor-bearing mice. Consistently, multi-color immunofluorescent staining showed, in both models, that combination treatment significantly promoted the infiltration of CD8^+^ T cells and cytotoxic IFN-γ^+^ CD8^+^ T cells, as compared to anti-PD-1 antibody did (**Fig. 6C**).

Furthermore, Foxp3^+^ CD25^+^ CD4^+^ T cells (Treg), defined as a suppressor in aberrant immune response, also play a role in the suppression of anti-tumor immunity 31. In the 4T1 tumor bearing mice, the population of Treg cell was apparently declined in the combination group, with respect to the control group (CGA + Anti-PD-1 vs. NS + IgG, p < 0.05). Interestingly, monotherapy with anti-PD-1 antibody increased the Treg cell population, suggesting that CGA might reverse this effect (CGA + Anti-PD-1 vs. NS + Anti-PD-1, p < 0.001) (**Fig. 6D**). In summary, CGA in combination with anti-PD-1 antibody promoted activation of the cytotoxic T cells, leading to tumor cell cytolysis and death. It appears that, with attenuation of the Treg cells in tumor tissue by CGA, the antitumor immunity of the anti-PD-1 antibody might be facilitated.

**Figure 7:**
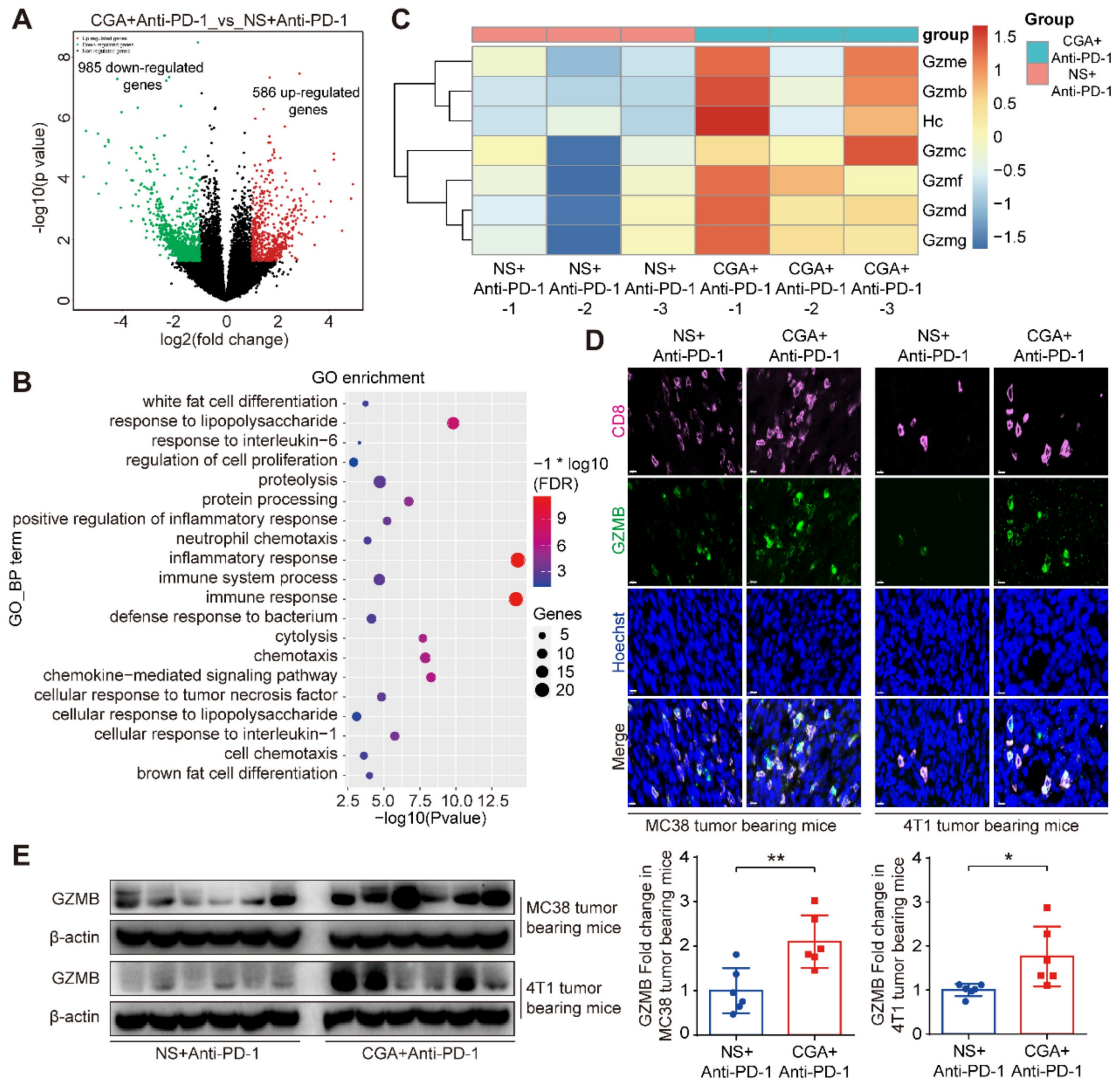
** CGA up regulated Granzyme B (GZMB) expression in tumor tissues**. The tumor tissues of the anti-PD-1 antibody monotherapy group (NS+Anti-PD-1, n = 3) and the CGA + anti-PD-1 antibody combination treatment group (CGA+Anti-PD-1, n = 3) from the 4T1 tumor bearing mice were collected. Total RNA of tumor tissues was extracted and the RNA sequence analysis was performed using “limma” and “DESeq” R packages. The differential expression genes (DEGs) were enriched in GO functions by DAVID database. **A**. DEGs comparison was shown in volcano plot with |log_2_FC| ≥ 1 and *p*-Value ≤ 0.05. **B**. The top 20 enrichment of GO biological process function terms. **C**. Heatmap of gene expression for cytolysis function of the NS+Anti-PD-1 and CGA+Anti-PD-1 group. Scale represents log transformed TPMs + 1, the red to blue represents the relatively gene expression from high to low. In the experiment groups, NS+Anti-PD-1-1, NS+Anti-PD-1-2, NS+Anti-PD-1-3, and CGA+Anti-PD-1-1, CGA+Anti-PD-1-2, CGA+Anti-PD-1-3 are the samples that were sequenced. **D-E**. CGA+Anti-PD-1 treatment up regulated the protein expression of GZMB in tumor tissues. Representative multi color immunofluorescent images stained of CD8 (pink) and GZMB (green) in tumor tissues (**D**). Scale bar. 10 μm. The protein expression of GZMB was tested by Western blot analysis (**E**). The results of GZMB were normalized to β-actin, as density ratio. Data are presented as mean ± SD (n = 6). Significant differences are indicated: *p< 0.05, **p< 0.01, vs NS+Anti-PD-1 group by Student's *t*-test. GZMB indicated granzyme B.

To further identify the relevant mechanisms, we performed RNA sequence analysis in tumor tissues from the mice treated with combination therapy or anti-PD-1 antibody alone. Differentially expressed genes (DEGs) analysis of limma R package 32 were used in the study. With respect to that of the anti-PD-1 antibody group, 586 up-regulated and 985 down-regulated genes were detected in total RNA of the combination group (**Fig. 7A**). Next, expression of the up-regulated genes went through enrichment analysis using DAVID Functional Annotation Tool 33. The top GO functions were presented in **Supplementary Fig. S12**. DEGs were mostly involved in biological processes such as immune response and cytolysis (**Fig. 7B**). Further analysis showed that the granzymes' genes were heavily affected by CGA and largely up-regulated in the combination group (**Fig. 7C**), as compared with that of the antibody monotherapy. Granzymes are secreted from CTLs or natural killer cells and plays an essential role for killing functions of cytotoxic T cells, leading to apoptosis or/and tumor cell cytolysis 34-36. Granzyme B, one of main cytolytic granule contents encoded by *Gzmb*, was up-regulated by CGA in the T-cell containing tumor tissues of the two tumor-bearing mice models (p < 0.01, p < 0.05) (**Fig. 7D and 7E**). Thus, CGA, in combination with anti-PD-1 antibody, could either enhance anticancer immunity of the infiltrated CD8+ T cells (via inhibiting PD-L1 expression in tumor) or accelerate tumor cell cytolysis (by enhancing T cell granzymes expression). The changes by CGA in the tumor microenvironment might promote the anticancer activity of anti-PD-1 antibody.

## Discussion

Immunotherapy with antibodies that block the PD-1/PD-L1 interaction has achieved great success in cancer treatment. However, the immune response in patients varies, and most of patients failed to benefit from immunotherapy. PD-L1 expression in tumor tissue is recognized as a mechanism and even a vital biomarker to predict anticancer immune response in patients 8, 9. Downregulation of PD-L1 expression in tumors might enhance the anticancer effect of immunotherapy 37, 38. In the present study, we show that cancer differentiation inducer CGA could reduce the expression of PD-L1 in cancer cells, thus protect tumor-infiltrated T cells from the PD-1/PD-L1 interaction caused T-cell death and so, enhance the therapeutic effect of the anti-PD-1 antibody. Therefore, combining CGA with anti-PD-1 antibodies might be a promising strategy to enhance the efficacy of immune checkpoint inhibitors.

Cancer cell differentiation represents a programmed intracellular synergistic course that shifts cancer cell from malignancy toward benign or even normal standing, characterized with reduced proliferation, metastasis, as well as immune checkpoint function. This biological process is complicated, and after trigging the program signal pathways and gene expression profiles in cancer cells are basically regulated. Reprogrammed by CGA, the reduced expression of PD-L1 could be an important sign of differentiation in cancer cells, and the pathway involves with, at least, IFN-γ/JAK/pSTAT1/IRF1/PD-L1.

IFN-γ, secreted by cytotoxic T cells and natural killer cells, is an essential molecule in either innate or adaptive immune response. As a double-edged sword, IFN-γ not only participates in the cytotoxic process of T cells but also induces PD-L1 expression in tumor cells, which eventually leads to tumor immune evasion 39. In this study, we found that CGA inhibited IFN-γ-induced PD-L1 expression in human melanoma cancer cells, triple-negative breast cancer cells, and ovarian cancer cells. As the stability of mRNA influences protein expression 28, we first detected the stability of PD-L1 mRNA and found that it was not disrupted by CGA treatment. Then, the activity of the PD-L1 promoter was detected as it is another important factor to express PD-L1 30, 40, 41. We found that the activity of the PD-L1 promoter was inhibited by CGA. The JAK-pSTAT1-IRF1 pathway is associated with IFN-γ induced upregulation of PD-L1^30^, and at the up-stream of the pathway IFN-γ binds to the IFN-γ receptor (IFNGR1 and IFNGR2), followed by activation of the JAK-pSTAT1-IRF1 path. As a transcriptional factor, IRF1 binds with the promoter sequence of PD-L1 gene and thus activates PD-L1 expression. We first tested the expression of IFNGR1 and IFNGR2, and found no change after CGA treatment. However, the overexpression of transcriptional factor IRF1 abolished the inhibitory effect of CGA on the activity of PD-L1 promoter, indicating IRF1 a main mechanism for CGA's activity. Previous studies have suggested that STAT1 is phosphorylated prior to IRF1 activation in the IFN-γ induced PD-L1 stimulation pathway 30. Here, we discovered that CGA downregulated the phosphorylation of STAT1, agreeing with the previous report. Thus, CGA might inhibit PD-L1 expression in tumor cells via suppression on the STAT1 phosphorylation-IRF1-PD-L1 pathway. In addition, as IRF1 has been reported to bind DNA of STAT1 and to promote phosphorylation of STAT1^42^, downregulation of IRF1 by CGA could further attenuate STAT1 phosphorylation and strengthen the inhibitory effect on PD-L1. Also, p-STAT1 is a transcriptional factor that directly binds to PD-L1 promoter 43; thus, inhibition of STAT1 phosphorylation by CGA might add extra suppression to the PD-L1 expression 44. Indeed, in the tumor/immunity cell co-culture system, the susceptibility of A375, MDA-MB-231, and SK-OV-3 cells towards the activated Jurkat E6 cells was increased by CGA. These results suggested that inhibition of PD-L1 expression in tumor cells by CGA could increase the activity of T cells and benefit the blockade of the tumor-immune checkpoint function. In brief, through the combination therapy, the expressions of PD-L1 and IRF1 were significantly reduced in tumor tissues by CGA, and the interaction between PD-1 and PD-L1 was blocked by the antibody, thus the T cell mediated antitumor immunotherapy was promoted. In the *in vitro* experiments, extension of CGA treatment time increased the inhibition of PD-L1 expression in tumor cells, suggesting an enhanced suppression along with the progress of cell differentiation.

Tumor response to the immunotherapy has been ranged from high to non. Thus, they were classified as “Hot”, “Cold”, and “Altered” 45, 46. The definition of “Hot”, “Cold”, and “Altered” tumor is mainly associated with the number of T cell infiltrated in tumor tissues 10. Cancers with high T cell infiltration in tumor microenvironment are considered as “Hot”, and those with low infiltration are “Cold”; tumors that have T cell infiltration in invasion margin and/or display low CD8^+^ and immunosuppressive subtype are within the “Altered” group 10. In the present study, two xenograft models were selected for the investigation, of which murine MC38 was recognized as a “Hot” tumor model, and 4T1 one as “Altered” model, according to the tumor immune response to the anti-PD-1 antibody and the level of infiltrated CD8^+^ T cells 47 (**Fig. 4 and 6**). The anti-tumor effect was evaluated for CGA, anti-PD-1 antibody and their combination; the results showed that combination of CGA with anti-PD-1 antibody had a better therapeutic effect than monotherapy, causing additional suppression on tumor growth in either “Hot” or “Altered” xenograft models (31.1% therapeutic effect increasing for MC38, p < 0.05; 24.5% therapeutic effect increasing for 4T1, p < 0.01).

The inhibitory effect on tumor growth in the MC38 (“Hot” model) by the combination was much more significant than that in the 4T1 one (“Altered”). The explanation for this difference could be associated with the T cells localized in tumor tissues, in which the number, cell types and activity of infiltrated T cells could influence antitumor immunity in the microenvironment 48, 49. In general, in the “Hot” model, the number and activity of T cells was higher than that in the “Altered” and “Cold” models, thus produced strong immune response against cancer 10, 50. In the present study, the difference of infiltrated T cells (CD3^+^ CD8^+^) in tumor tissues of two tumor bearing models were observed (**Fig. 6A-B**), which might contribute to the distinctive tumor suppression effect seen in the two models.

Cytotoxic T lymphocytes (CTLs) are main effective lymphocytes in anti-tumor immunity. The IFN-γ^+^ CD8^+^ T cells play an important role in cytotoxicity towards tumor cells, and thus the proportion of this T cell subset is recognized as a positive indicator for anti-tumor immunity 51. In learning the influence of CGA on CTLs, we found that the CD8^+^ CD3^+^ T-cell population, which was a subset of CTLs, increased in both the combination group and CGA or anti-PD-1 antibody monotherapy group. Next, we analyzed the proportion of infiltrated IFN-γ^+^ CD8^+^ T cells in tumor tissues. Our data showed that the proportion of IFN-γ^+^ CD8^+^ T cells was increased in the combination group in the two murine tumor models; for the MC38 tumor-bearing mice, improvement of the IFN-γ^+^ CD8^+^ T cells was more significant than monotherapy (**Fig. 6B;** 2.77-fold upregulated with respect to the NS + Anti-PD-L1 group, p < 0.001, respectively), agreeing with its therapeutic efficacy (**Fig. 4A-D**). These results suggested that CGA could increase the number of cytotoxic T cells in the tumor microenvironment. At the same time, regulatory T cells (Treg), which is a suppressor in antitumor immunotherapy and correlates with poor prognosis in cancers 31, were also investigated. Monotherapy with anti-PD-1 antibody increased the number of Treg cells in tumor tissues; however, the undesired increase was reversed by CGA in combination therapy for the 4T1 tumor model (**Fig. 6D**). For the increase of Treg cells in the antibody monotherapy, it is possible that the PD-1 expressed Treg cells were protected by anti-PD-1 antibody via blocking the interaction between PD-1 and PD-L1 in tumor tissues 52; but for the inhibitory effect on the Treg cells by CGA that we have seen in the combination group, more investigation is needed. We should mention here that CGA did not change systemic T cell immunity in animal experiments 53, 54, but might be effective in regulating macrophages and cytokines 26, 55-59.

Granzymes are a group of proteases secreted by CTLs and natural killer cells and executes the cell-killing effect with help from perforin 34, 36, 60. Granzyme B (GZMB) is one of the main molecules in the granzyme family and has been reported to have a positive relationship with clinical outcome after PD-1 blockade treatment 61. In this study, we found that granzymes genes in tumors were upregulated in combination treatment, as compared to the anti-PD-1 antibody monotherapy (**Fig. 7**). This modulation was further verified at the protein level in the tumor tissues of the two models. We consider it supportive evidence for the enhanced T-cell activation and/or T- cell counts in tumor microenvironment.

**Figure 8:**
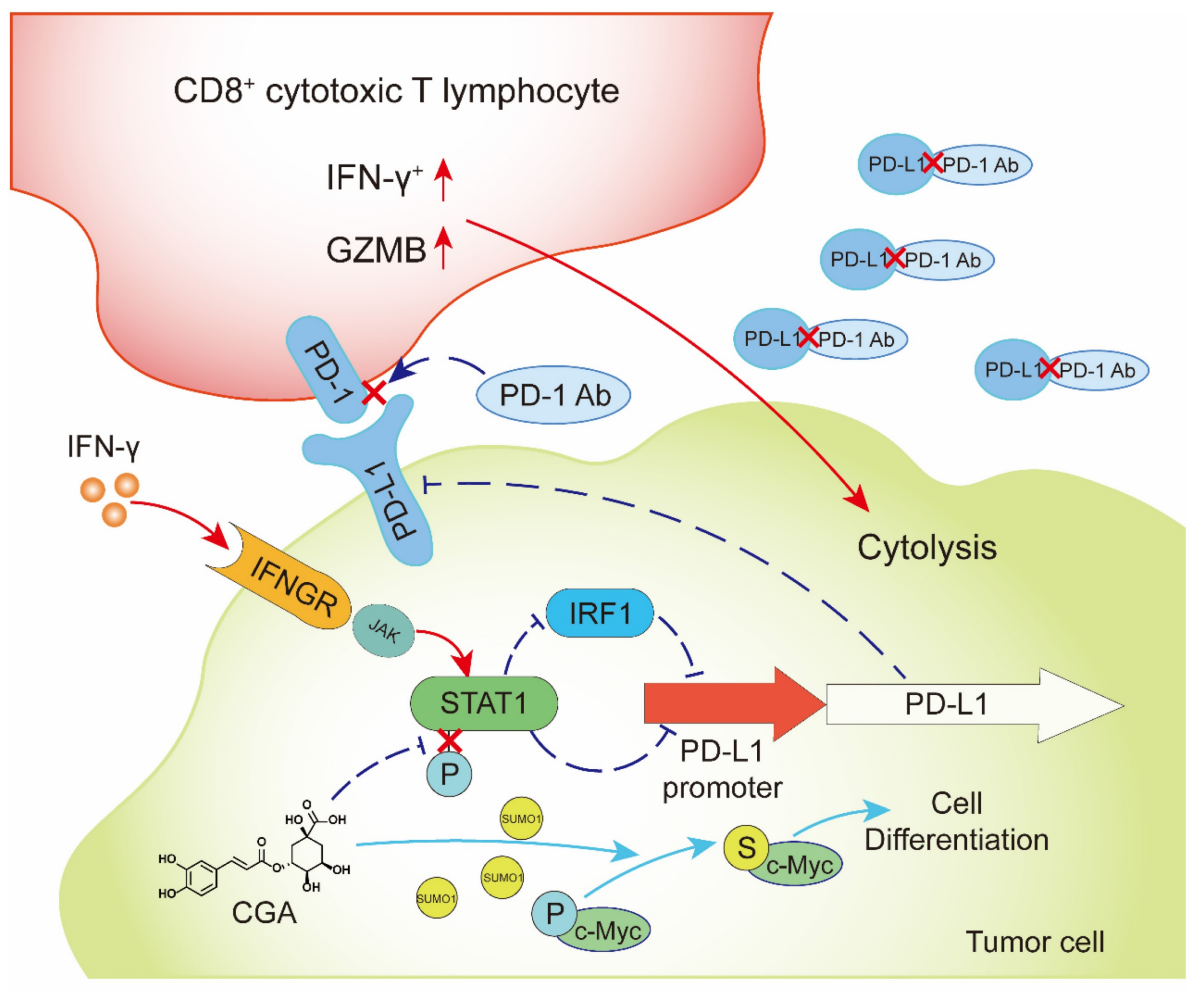
** CGA inhibited PD-L1 expression induced by IFN-γ *via* inhibition of STAT1 phosphorylation and enhanced tumor immunotherapy**.

In summary, CGA is a known cancer differentiation inducer (CDI) and its Phase II clinical trial for glioma is now at its late stage in China. We show here that CGA suppressed tumor cell PD-L1 expression induced by IFN-γ, through inhibiting IFN-γ/JAK/pSTAT1/IRF1/PD-L1 pathway. Thus, CGA promotes T-cell activity in tumor microenvironment and enhances anti-tumor effect when combined with anti-PD-1 antibody (**Fig. 8**). As a CDI, CGA could be a safe immune enhancer to improve the anticancer efficacy if used with anti-PD-1 antibodies.

## Materials and Methods

### Materials

Chlorogenic acid (CGA) was acquired from the Jiuzhang Biochemical Engineering Science and Technology Development Co., Ltd. (Chengdu, Sichuan, China), which with a purity of more than 99%. CGA was dissolved in normal saline (NS) at a concentration of 100 mM as a stock solution for *in vitro* experiment. Interferon-Gamma (IFN-γ) (PeproTech #315-05) was dissolved in 0.3% BSA solution at a concentration of 10 μg/mL as a stock solution. PD-1 neutralizing antibody and isotype control antibody were purchased from BioXcell, and diluted at a concentration of 2 mg / ml using Dilution Buffer (BioXcell).

### Cell culture

Human melanoma cell line A375, ovarian cancer cell line SK-OV-3, lung large cell carcinoma cell NCI-H460, squamous cell carcinoma of head and neck cell CAL-27 were purchased from the National Infrastructure of Cell Line Resource (Beijing, China). Breast cancer cell line MDA-MB-231, human T lymphocyte leukemia cell Jurkat E6, human embryonic kidneys cell HEK-293T, and murine colon carcinoma cell MC38 were the storage of our laboratory. Murine breast cancer cell line 4T1 was from the American Type Culture Collection (ATCC, MD, USA).

The A375, MDA-MB-231 and CAL-27 were cultured in the Dulbecco's Modified Eagle's Medium (Invitrogen, CA, USA) with 10% fetal calf serum (FBS, Invitrogen), penicillin (100 U/mL) and streptomycin (100 μg/mL) (Invitrogen). The SK-OV-3 was cultured in the McCoy's 5A Media (Invitrogen) with 10% FBS, penicillin and streptomycin (P/S, Invitrogen). The NCI-H460, Jurkat E6, MC38 and 4T1 were cultured in the RPMI-1640 medium (Invitrogen) supplemented with 10% FBS and P/S. All cells were cultured at 37 °C and 5% CO_2_.

The A375, SK-OV-3, MDA-MB-231, NCI-H460 and CAL-27 cells were seeded into 12-well plate with full growth medium at 2 × 10^5^ cell per well for 24 h before treatment and cultured overnight. The cells were treated with IFN-γ (10 ng / mL) and different concentrations of CGA (0 - 200 μM) for 48 h.

### Plasmids

The plasmid of pGL3-PD-L1-promotor was constructed by the vector of pGL3-Basic. The nucleotide fragment of the promotor of PD-L1 was amplified by polymerase chain reaction (PCR) from A375 genomic DNA with the primers of 5′- TAGAAGTTCAGCGCGGGATAATACTTAA -3′ and 5′- CAGCGAGCTAGCCAGAGATACTGGGC -3′ and cloned into multicloning site (MCS) with *Sac*I and *Xho*I of the pGL3-Basic vector as previously described.

The plasmid of IRF1 expression was built from the vector of pCDNA3.1-MYC-HIS-C while the inserted fragment was synthesized by TsingKe Biological Technology Co. (Beijing, China) and connected to the vector by the internal restriction site of *BamH*I. pRL-TK, pCDNA3.1/myc-HIS-C, and pGL3-Basic plasmids were the storage of laboratory.

### Dual luciferase reporter assay

The A375, SK-OV-3 and MDA-MB-231 cells were seeded into 96-well plates with full growth medium at 5 × 10^3^ cells per well the day before transfection and cultured overnight until they reached 70-80% confluence. The transfection of pGL3-PD-L1-promotor (100 ng per well) and the normalization plasmid of pRL-TK (3 ng per well), were carried out using Lipofectamine 2000 (Invitrogen) according to the manufacturer's protocol. Six hours in culture after transfection, CGA with different concentrations (0 - 200 μM) and IFN-γ (10 ng / mL) were added into the wells. The transfection was finished after 48 h and then the cells were treated by 100 μL of Passive Lysis Buffer (PLB). The lysate was transferred and immediately measured using the Dual-Luciferase ^®^ Report Assay System and a GloMax 96 Microplate Luminometer (Promega). Data were calculated by Relative luciferase units (RLUs), RLUs from firefly luciferase signal were normalized by RLUs from Renilla signal. All assays were executed for three times.

### Western Blot

Western blotting was performed as previously described 28. The primary antibodies for PD-L1 (CST #13684), IRF1 (CST #8478), STAT1 (CST #14994), p-STAT1 (CST #9167), GZMB (CST #17215), β-actin (CST #4970) and the matching horseradish peroxidase (HRP) conjugated secondary antibodies were purchased from Cell Signaling Technologies (CST). Immobilon Western Chemiluminescent HRP Substrate (Millipore, Billerica) was added, and the bands were imaged with the Chemidoc XRS+ electrophoretic imaging system (Bio-Rad). Density scanning of each protein band was performed using Image Lab software (Bio-Rad). The results were normalized to β-actin.

### Quantitative real-time PCR (RT-PCR)

Total RNA of cells was extracted using TRIzol ® Plus RNA Purification Kit (Invitrogen) and was reverse transcribed into cDNA by High-Capacity cDNA Reverse Transcription Kit (Applied Biosystems). The RT-PCR experiment was performed by power SYBR Green reagent (Applies Biosystems) and 7500 Fast Real-Time System (Applied Biosystems). The mRNA expression of genes was normalized with the housekeeping gene GAPDH. The primer sequences used are listed in **Supplementary Table S1**.

### Animal Models

Four-week-old male C57BL/6N mice and female BALB/c mice were purchased from the Charles River (Beijing. China) and housed in a temperature-controlled room at 20 ± 1 °C on a 12 h light/dark cycle. All experimental procedures on animals were authorized by the ethics committee of the Institute of Materia Medica, Chinese Academy of Medical Sciences and Peking Union Medical College (Beijing, China). For subcutaneously inoculated, 2 × 10^5^ cells of MC38 were injected under the skin of male mice back and 1 × 10^5^ cells of 4T1 were injected under the skin which was near the mammary gland of the female mice. Three days after implantation, the mice were randomly divided into four groups (NS + IgG, NS + Anti-PD-1, CGA + IgG, and CGA + Anti-PD-1). CGA (50 mg / kg) treatment was performed every day and lasted almost three weeks, while neutralizing antibodies (200 ug each time) were injected at the 3^rd^, 7^th,^ and 10^th^ day. Tumor size was measured every two days, and the volume of the tumor was calculated with the formula: V = 1/2 × length × width^2^. After sacrificing the mice, the serum and the tumor tissue samples were collected.

### Luminex Multiplex Assay

The cytokine of the serum samples was detected by Luminex Multiplex Assay with Bio-Plex Pro Mouse Cytokine 23-plex Assay (Bio-Rad) according to the manufacturer's protocol and analysis with Bio-Plex Manager software (Bio-Rad).

### Immunofluorescence

Three tumor cells grown on cover glasses and were fixed by 4% paraformaldehyde. After washing with cold PBS, the cell membranes were permeabilized through 0.5% Triton X-100. Next, the samples were blocked with normal goat serum, and incubated with diluted antibody solution at 4 °C overnight. After a wash in cold PBS, the samples were incubated with diluted secondary antibodies at room temperature. After wash, the cells were stained with DAPI. Analyze the signal under a fluorescence microscope (Olympus).

### Multiple Color Immunofluorescent

Tumor tissues were fixed in 4% paraformaldehyde for 24 h and paraffin embedded. Longitudinal cross sections of the mouse tumor tissue from these blocks were obtained and deparaffinization was performed by warming the slides at 65 °C for 30 min. The slides were then immersed in xylenes and next immersed in 100%, 95% then 70% ethanol for 15 min each. The slides were washed under running tap water and then incubated in a 1% hydrogen peroxide/methanol solution for 10 min. The slides were rinsed with distilled water and rinsed in PBST (Phosphate-Buffered Saline containing 0.05% Tween-20), then incubated with an anti-mouse-PD-L1 (Abcam #ab213480), anti-mouse IRF1 (CST #8478), anti-mouse CD8 (Abcam #ab217344), anti-mouse IFN-γ (Abcam #ab216642), anti-mouse-Cl. Caspase 3 (Proteintech #19677-1-AP), anti-mouse-PCNA (CST #2586), anti-mouse GZMB (Abcam #ab255598) at room temperature for 1 h. The slides were rinsed with PBST and incubated with HRP-labeled Polymer AntiRabbit (CST) at room temperature for 30 min. After a rinse with PBST, the slides were incubated with TSA (Tyramide signal amplification) for visualization. Next, the slides were washed with distilled water and then dehydration with 75%, 95% and 100% alcohol. Last, put the slides in xylene solution for 20 min and treated with neutral gum.

### Tumor infiltrating lymphocyte isolation

Tumor tissues were freshly collected from animal model and cut into small pieces, and then grinded through a 70 μm filter by a blunt end of syringe. The cell suspension was slowly added to Ficoll reagent and centrifuge at 2, 000 rpm horizontally for 30 min. Tumor infiltrating lymphocyte (TIL) were enriched in the second phase.

### Flow cytometry

Tumor infiltrating lymphocyte (TIL) was suspended by MACS buffer and blocked with anti-CD16/32 antibodies (BD, #553141) for 10 min at 4 °C. Next, cells were incubated with surface marker antibodies CD45 (BD #550994), CD3 (BioLegend #100320), CD8 (BD #553030), CD4 (BioLegend #100516), CD25 (BioLegend #102006) for 30 min. After that, cells were stimulation with PMA (50 ng/mL), Ionomycin (1 μg/mL), Brefeldin A (1 μl/mL) and GolgiStop (1 μl/mL) for 5 h before fixation and permeabilization. After fix and permeabilization, cells were incubation with the intracellular antibodies IFN-γ (BD #561479) and Foxp3 (Invitrogen #25-4777-42) for 30 min at 4 °C. TIL was washed three times with MACS buffer and analyzed by FACSVerse flow cytometer with FlowJo software.

### RNA-seq and data analysis

Total RNA of tumor tissues was extracted by using TRIzol ® Plus RNA Purification Kit (Invitrogen, USA). High-quality (Agilent Bioanalyzer RIN > 7.0) total RNA was applied for preparation of sequencing libraries using the NEBNext Ultra RNA Library Prep Kit. A total of 2-3 μg of riboRNA-depleted total RNA was used to construct the eukaryotic strand-specific library and sequenced by an Illumina Hiseq-PE150. The data were normalized and analyzed with “limma” and “DESeq” R package. DEGs were performed function enrichment by DAVID database.

### Statistical analysis

Statistical analysis was performed using GraphPad Prism 6 (CA). All data are presented as the mean ± SD unless otherwise stated. Student's *t*-test and One-way ANOVA test were used, unless otherwise stated. We considered *P* < 0.05 to be statistically significant.

### Data Availability

The data generated in this study are available upon request from the corresponding author.

## Supplementary Material

Supplementary figures and table.

## Abbreviations

CGA: chlorogenic acid
IFN-γ: interferon-γ
CDI: cancer differentiation inducer
ICI: immune checkpoint inhibitor
PD-1: programmed cell death-1
PD-L1: Programmed cell death 1 ligand 1
IRF1: interferon regulatory factor 1
STAT1: signal transducer and activator of transcription 1
p-STAT1: phosphorylated signal transducer and activator of transcription 1
CTLA-4: cytotoxic T lymphocyte-associated protein 4
PCNA: proliferating cell nuclear antigen
CTL: cytotoxic T lymphocyte
GZMB: granzyme B
