# Supplementary material for: Cancer Differentiation Inducer Chlorogenic Acid Suppresses PD-L1 Expression and Boosts Antitumor Immunity of PD-1 Antibody
Source: Int J Biol Sci. 2024 Jan 1;20(1):61–77. doi: 10.7150/ijbs.83599 (PMC10750284; doi:10.7150/ijbs.83599)
Supplement: Supplementary file 1 — Supplementary figures and table. [file ijbsv20p0061s1.pdf]

Cancer Differentiation Inducer Chlorogenic Acid Suppresses PD-L1 Expression and Boosts Antitumor Immunity of PD-1 Antibody

Rui Li[1, #], Yun Zhan[2, #], Xiao Ding[3], Jinjin Cui[1], Yanxing Han[2], Jinlan Zhang[2], Jie Zhang[4], Wenbin Li[5], Lulu Wang[1, *], Jiandong Jiang[1, 2, *]

1. Institute of Medicinal Biotechnology, Chinese Academy of Medical Sciences and Peking Union Medical College, Beijing, 100050, China.
2. State Key Laboratory of Bioactive Substances and Function of Natural Medicine, Institute of Materia Medica, Chinese Academy of Medical Sciences and Peking Union Medical College, Beijing, 100050, China.
3. State Key Latoratory of Phytochemistry and Plant Resource in West China, Kunming Institute of Botany, Chinese Academy of Sciences, Kunming, Yunnan, 650201, China.
4. Jiuzhang Biochemical Engineering Science and Technology Development Co., Ltd, Chengdu, Sichuan, 610041, China.
5. Department of Neuro-oncology, Cancer Center, Beijing Tiantan Hospital, Capital Medical University, Beijing 100070, China.

Supplementary Data

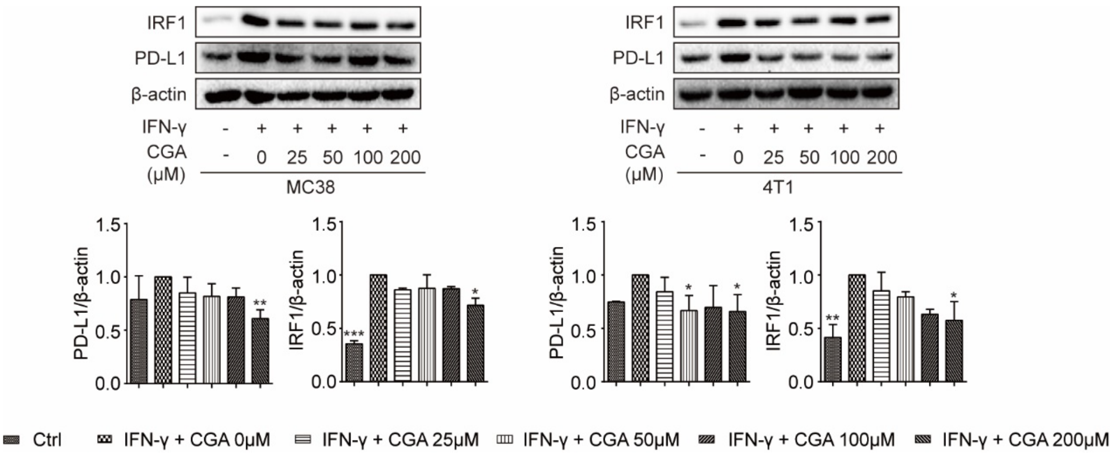

Fig. S1. CGA suppressed PD-L1 expression of murine cancer cells. CGA diminished PD-L1 expression induced by IFN-γ in a variety of cancer cells. Murine colon carcinoma cell MC38 and murine breast cancer cell 4T1 were treated with IFN-γ (10 ng/mL) and CGA (0, 25, 50, 100, 200 μM) for 48 h at 37˚C in 5% CO2. The protein expression of PD-L1 and IRF1 was tested using Western blot analysis. The results were normalized to β-actin as density ratio. Significant differences are indicated: *p< 0.05, **p< 0.01, ***p< 0.001, ns, not significant, vs. 0 μM group by One-way ANOVA test. CGA indicated chlorogenic acid; IFN-γ, interferon-γ; IRF1, interferon regulation factor 1.

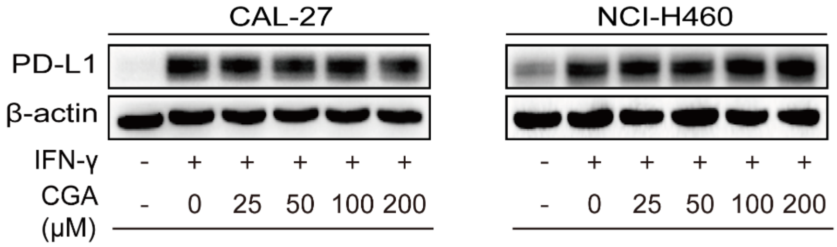

Fig. S2. CGA did not influence PD-L1 expression in human lung large cell carcinoma cell NCI-H460 and squamous cell carcinoma of head and neck cell CAL-27. Cancer cells treated with IFN-γ (10 ng/mL) and CGA (0, 25, 50, 100, 200 μM) for 48 h at 37˚C in 5% CO2. The protein expression of PD-L1 was tested using Western blot analysis. The results were normalized to β-actin as density ratio.

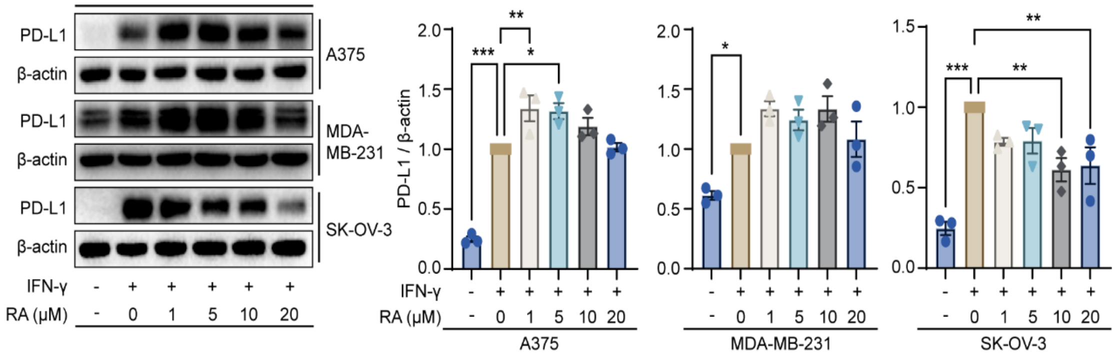

Fig. S3. RA regulated the expression of PD-L1 induced by IFN-γ in A375, MDA-MB-231, SK-OV-3 cell lines. Human melanoma cell line A375, ovarian cancer cell line SK-OV-3, and breast cancer cell line MDA-MB-231 were treated with IFN-γ (10 ng/mL) and RA (0, 1, 5, 10, 20 μM) for 48 h at 37˚C in 5% CO2. The protein expression of PD-L1 was tested using Western blot analysis. The results were normalized to β-actin as density ratio. Data are presented as mean ± SEM (n = 3). Significant differences are indicated: *p< 0.05, **p< 0.01, ***p< 0.001, ns, not significant, vs. 0 μM group by One-way ANOVA test.

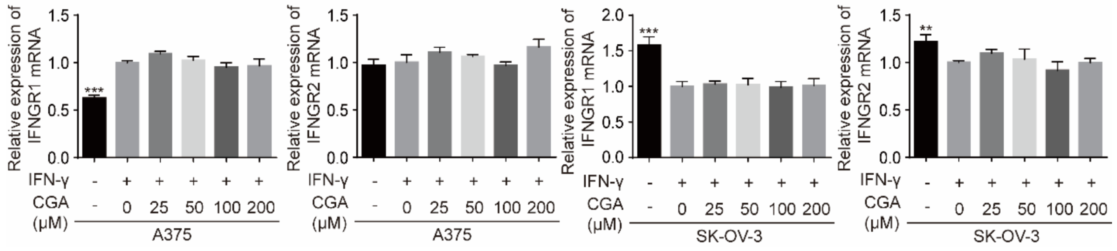

Fig. S4. CGA treatment did not inhibit IFNGR1 and IFNGR2 expression. Human melanoma cell line A375 and ovarian cancer cell line SK-OV-3 were treated with IFN-γ (10 ng/mL) and CGA (0, 25, 50, 100, 200 μM) for 48 hrs at 37˚C in 5% CO2. The mRNA expression of IFNGR1 and IFNGR2 was evaluated by RT-PCR. The results were normalized to GAPDH. Significant differences are indicated: **p< 0.01, ***p< 0.001, ns, not significant, vs. 0 μM group by One-way ANOVA test.

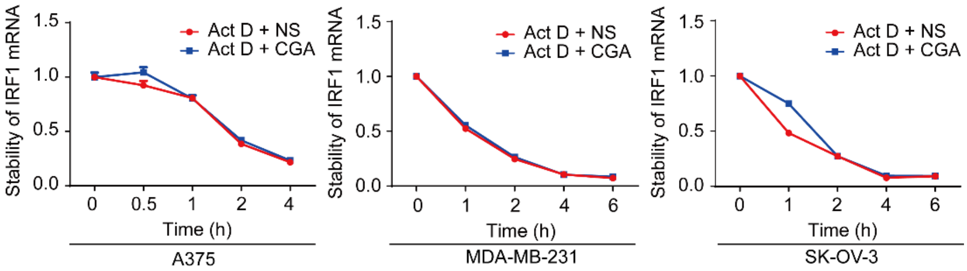

Fig. S5. The stability of IFR1 mRNA was not changed by CGA treatment. The cells were treated with IFN-γ (10 ng/mL) and CGA (200 μM). Act D (5 μM) were added to disrupt the stability of IRF1 mRNA. The mRNA of PD-L1 was evaluated by RT-PCR at 0, 1, 2, 4, 6 hrs in SK-OV-3 and MDA-MB-231 cell lines while at 0, 0.5, 1, 2, 4 hrs in A375 cell line. The results were normalized to GAPDH.

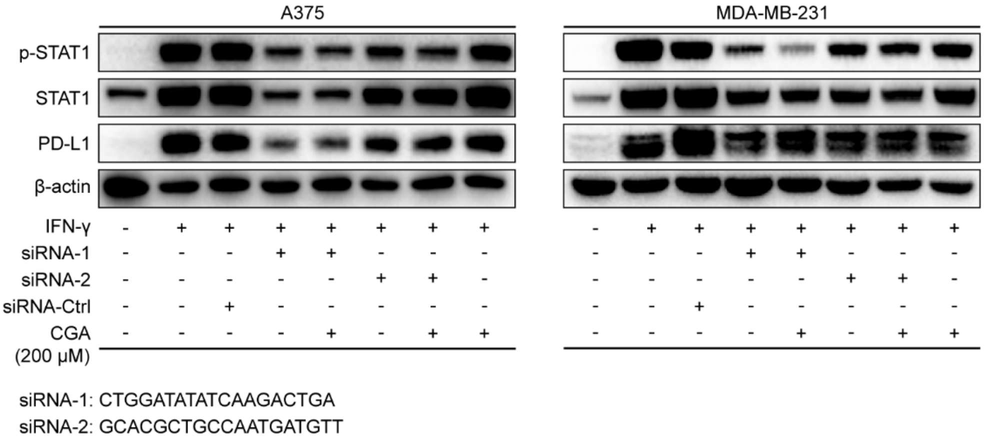

Fig. S6. Knocking down STAT1 by siRNAs in A375 and MDA-MB-231 cell lines. A375 and MDA-MB-231 were transfected with siRNA1, siRNA2, or siRNA-Ctrl (100 nM per well) and treat with IFN-γ (10 ng/ml) and CGA (200 μM) for 48 h at 37°C in 5% CO2. The protein expression of PD-L1 was tested using Western blot analysis.

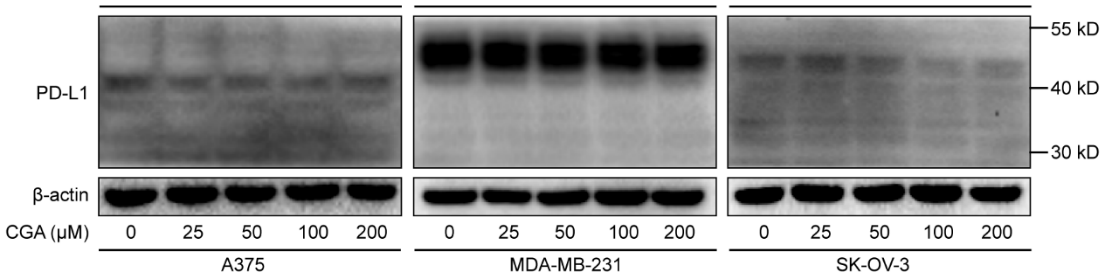

Fig. S7. **The regulation of CGA for the basal level of PD-L1 in A375, MDA-MB-231, SK-OV-3 cell lines.** A375, MDA-MB-231, and SK-OV-3 were treated with CGA (0, 25, 50, 100, 200 μM) for 48 h at 37˚C in 5% CO2. The protein expression of PD-L1 was tested using Western blot analysis.

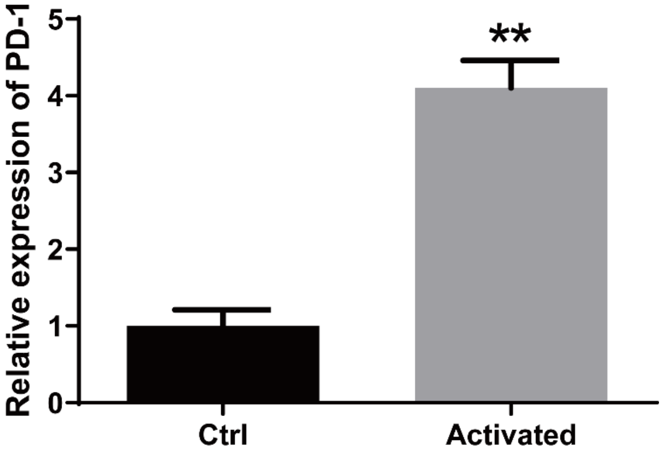

Fig. S8. The PD-1 expression was upregulated in activated Jurkat E6 T cells. Human T lymphocytic leukemia cells Jurkat E6 were treated with anti-CD3 antibody (100 ng/mL) and anti-CD28 antibody (100 ng/mL) for 24 hrs at 37˚C in 5% CO2. The mRNA of PD-1 was evaluated by RT-PCR. The results were normalized to GAPDH. Significant differences are indicated: **p< 0.01, vs. Ctrl group by Student's t-test.

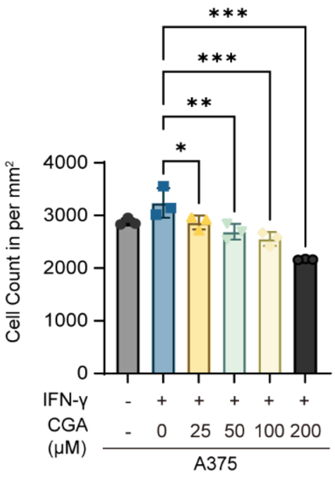

Fig. S9. The cell counts in per mm2 of the co-culture system for Jurkat E6 and three cancer cells. Data are presented as mean ± SEM (n = 3). Significant differences are indicated: *p< 0.05, **p< 0.01, ***p< 0.001, ns, not significant, vs. 0 μM group by One-way ANOVA test.

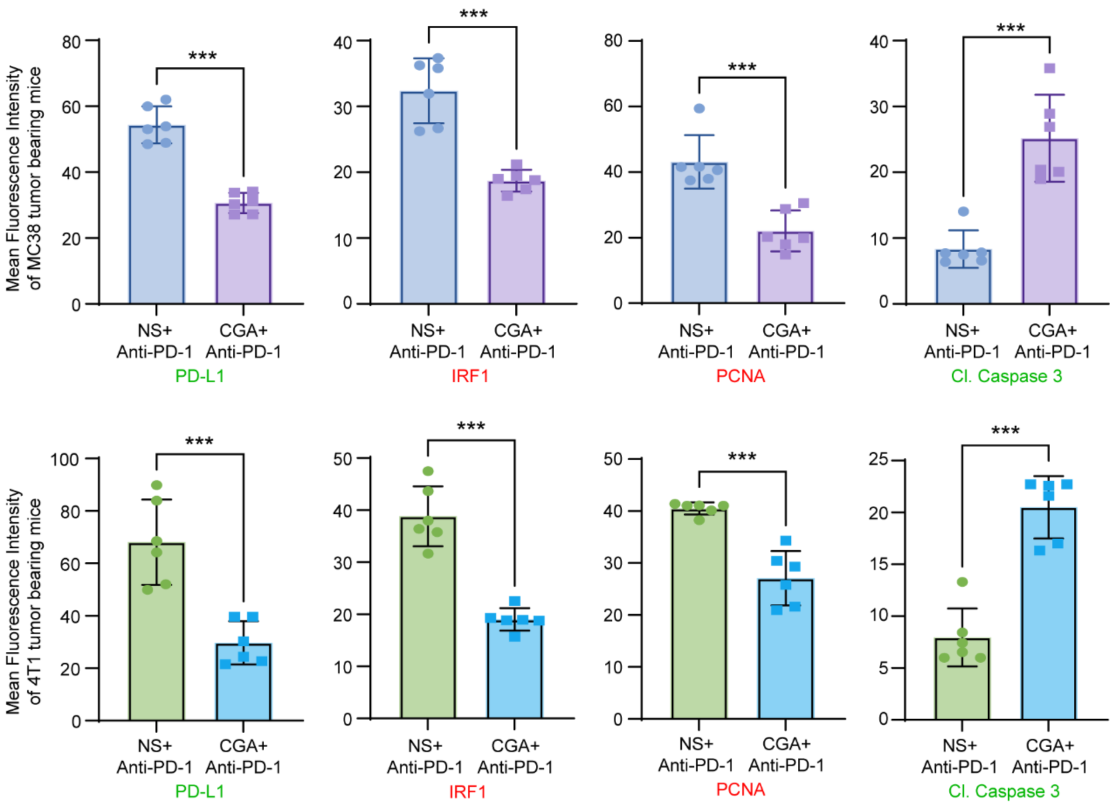

Fig. S10. The quantified mean fluorescence intensity of PD-L1, IRF1, PCNA, and Cl. Caspase 3 in multi-color immunofluorescence assay. The tumor tissues were

collected from mice (MC38 and 4T1 tumor bearing mice) in anti-PD-1 antibody monotherapy group (NS+Anti-PD-1) and CGA+Anti-PD-1 antibody combination treatment group (CGA+Anti-PD-1). Significant differences are indicated: **$p < 0.01$, vs. Ctrl group by Student's *t*-test.

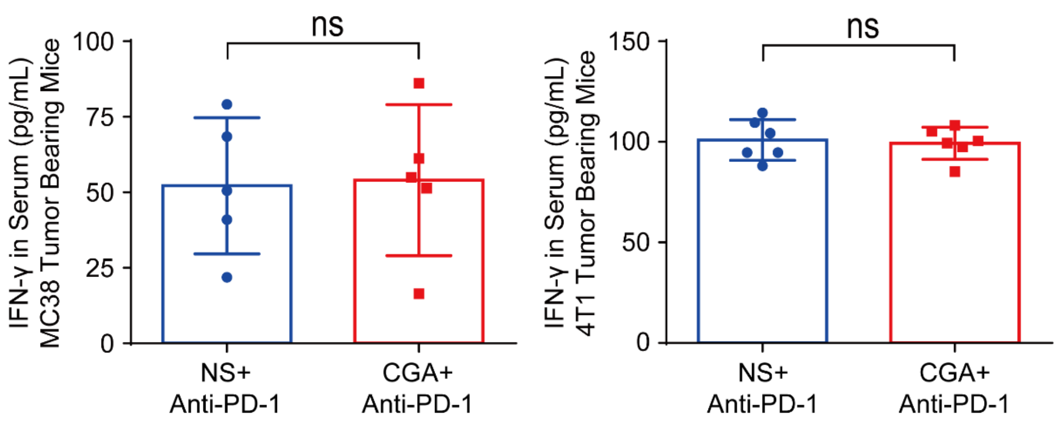

Fig. S11. CGA combined anti-PD-1 antibody therapy did not influence the serum IFN-γ concentration. The serum was collected from mice in anti-PD-1 antibody monotherapy group (NS+Anti-PD-1) and CGA+Anti-PD-1 antibody combination treatment group (CGA+Anti-PD-1). The serum IFN-γ concentration was detected by Luminex Multiplex Assay. Data are presented as mean ± SD (n = 5 or 6). Significant differences are indicated: ns, not significant, vs. NS + Anti-PD-1 group by Student's t-test.

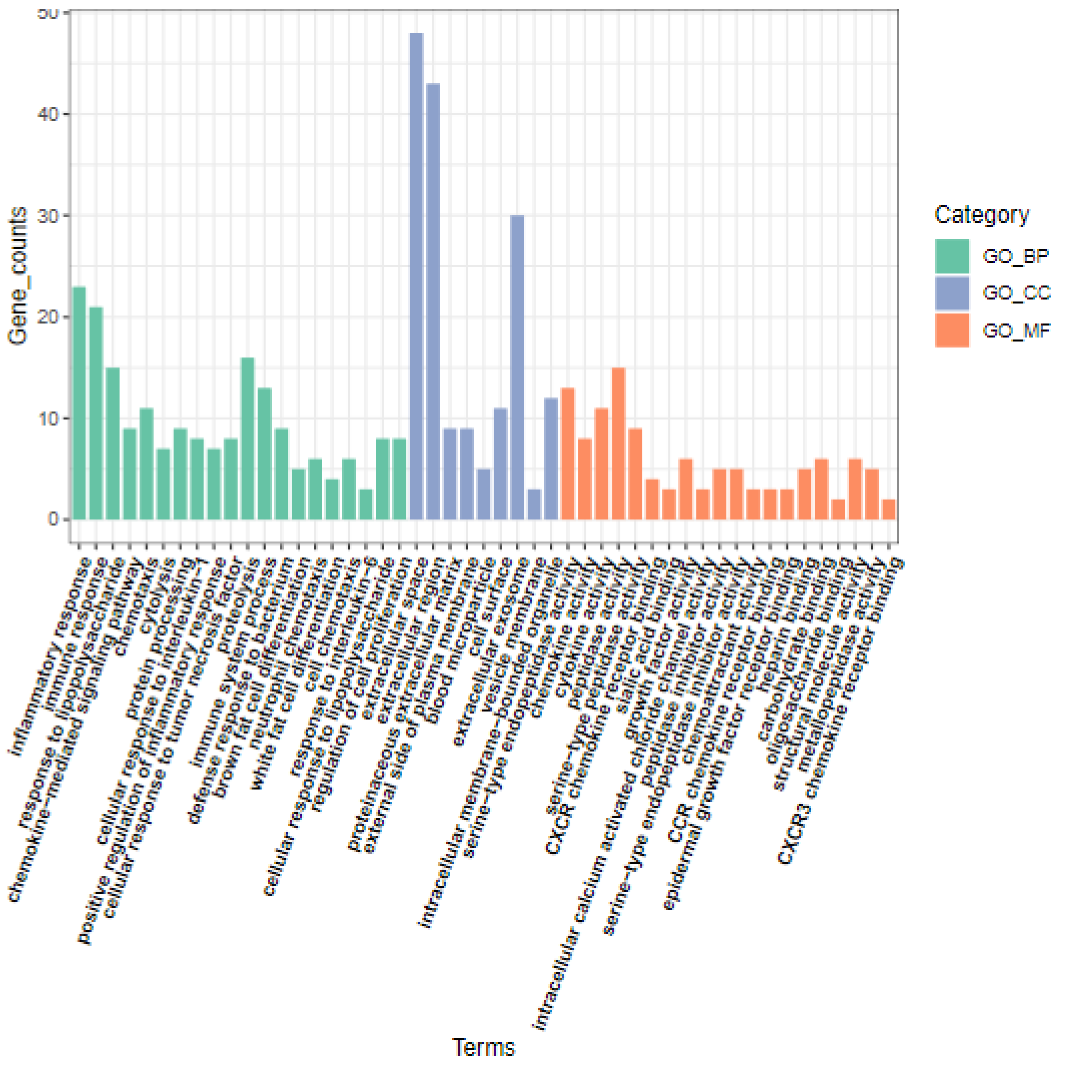

Fig. S12. DEGs enrichment in the top GO functions. The tumor tissues of the anti-PD-1 antibody monotherapy group (NS+Anti-PD-1, n = 3) and the CGA + anti-PD-1 antibody combination treatment group (CGA+Anti-PD-1, n = 3) from the 4T1 tumor bearing mice were collected. Total RNA of tumor tissues was extracted and the RNA sequence analysis was performed using “limma” and “DESeq” R packages. The differential expression genes (DEGs) were enriched in GO functions by DAVID database.

Table S1. Primer sequences for RT-PCR

Gene name	Forward	Reverse
homo		
CD274 (PD-L1)	CCTACTGGCATTTGCTGAACG	AGACAATTAGTGCAGCCAGGT
IRF1	GATGCCTGTTTGTTCCGGAG	CCTCGATATCTGGCAGGGAG
IFNGR1	GGCAGCATCGCTTTAAACTC	GGAGGTGGGGGCTTTTATTA
IFNGR2	CATCCTGATCTCCGTGGGAA	GACGTCATCCTTTGGTGAGC
GAPDH	GGTGAAGGTCGGAGTCAACG	TGGGTGGAATCATATTGGAACA
murine		
Cd274	GACGCCTCACTTGCTCATTA	CAGAGCTAATGGGCTCCTTC
Gapdh	CTCCCACTCTTCCACCTTCG	TAGGGCCTCTCTTGCTCAGT
